# Association of Optimal Blood Pressure With Critical Cardiorenal Events and Mortality in High-Risk and Low-Risk Patients Treated With Antihypertension Medications

**DOI:** 10.1001/jamanetworkopen.2019.9307

**Published:** 2019-08-23

**Authors:** Hae Hyuk Jung

**Affiliations:** 1 Department of Medicine, Kangwon National University Hospital, Kangwon National University School of Medicine, Chuncheon, South Korea

## Abstract

**Importance:**

There are few studies comparing the optimal level of treated blood pressure (BP) between high- and low-risk patients.

**Objective:**

To examine whether optimally treated BP is different according to risk status.

**Design, Setting, and Participants:**

Population-based cohort study using data from the National Health Information Database in Korea from 2002 to 2015 and 2006 to 2017. A total of 1 402 975 adults aged 40 to 79 years who had no known cardiorenal disease were included.

**Exposures:**

Systolic BP treated with antihypertensive medication.

**Main Outcomes and Measures:**

The yearly rates of critical cardiorenal events and all-cause death were estimated according to the levels of treated systolic BP and the presence of 5 risk factors (hypertension, diabetes, hyperlipidemia, proteinuria, and smoking).

**Results:**

During the study periods, 225 103 of 487 412 participants (54.0% male; median [interquartile range] age, 50 [44-59] years) in the primary cohort and 360 503 of 915 563 participants (50.1% male; median [interquartile range] age, 52 [46-60] years) in the secondary cohort received antihypertensive treatment. In total, 28 411 of 51 292 cardiorenal incidents and 33 102 of 72 500 deaths were noted in ever-treated participants. The absolute increase in cardiorenal and mortality risk associated with inadequately treated BP was greater in participants with multiple risk factors than in those with 1 or 0 risk factors. The hazard ratios for critical cardiorenal events increased as the treated systolic BP increased to more than 130 to 140 mm Hg. The hazard ratio for all-cause mortality for patients with 3 or more risk factors and treated systolic BP within the range of 110 to 119 mm Hg was 1.21 (95% CI, 1.07-1.37); 130 to 139 mm Hg, 1.04 (95% CI, 0.98-1.11); 140 to 149 mm Hg, 1.12 (95% CI, 1.05-1.20); 150 to 159 mm Hg, 1.21 (95% CI, 1.11-1.32); and 160 mm Hg or greater, 1.46 (95% CI, 1.32-1.62) compared with high-risk patients with BP of 120 to 129 mm Hg. For participants with 1 or 0 risk factors and treated systolic BP within the range of 110 to 119 mm Hg, the hazard ratio was 1.14 (95% CI, 1.07-1.22); 130 to 139 mm Hg, 0.97 (95% CI, 0.93-1.02); 140 to 149 mm Hg, 1.00 (95% CI, 0.91-1.09); 150 to 159 mm Hg, 1.06 (95% CI, 0.99-1.14); and 160 mm Hg or greater, 1.26 (95% CI, 1.15-1.37). However, when categorized using cardiovascular risk calculators, there was no consistent trend in mortality thresholds of BP across the risk score categories.

**Conclusions and Relevance:**

These results suggest that intensive BP control is appropriate for reducing all-cause mortality in addition to cardiorenal risk in higher- rather than lower-risk patients. However, caution may be required when determining BP targets using current risk calculators.

## Key Points

**Question:**

Is the optimal level of treated blood pressure (BP) associated with different rates of critical cardiorenal events and mortality in high- vs low-risk patients?

**Findings:**

In this population-based cohort study of more than 1 million adults in Korea, the absolute increase in cardiorenal and mortality risk with inadequately treated BP was larger in patients with more risk factors, and the mortality threshold of treated BP was left-shifted in patients with multiple vs 1 or 0 risk factors.

**Meaning:**

These findings support the need for individualized treatment of high BP considering more strict targets for higher-risk patients.

## Teaser

This population-based cohort study assesses whether the optimal level of treated blood pressure varies between patients with more vs fewer risk factors among adults in Korea.

## Introduction

Blood pressure (BP) lowering in patients with moderate to severe hypertension reduces cardiorenal and mortality risk. However, the optimal levels of treated BP in patients with diverse conditions have not been determined. Knowledge of the absolute benefits of treatments is generally important for individual therapeutic decisions, and the absolute benefit of BP lowering may be greater in high-risk than in low-risk patients, as suggested by previous studies.^1,2^ Accordingly, current guidelines recommend that clinicians consider intensive BP control if patients are at high risk on the basis of individualized assessments.^3,4,5^ Moreover, the 2017 American College of Cardiology/American Heart Association (ACC/AHA) guidelines recommend cardiovascular disease risk assessment using a risk score calculator to identify patients who require strict BP targets.^4^ However, the benefits of more intensive lowering of BP in patients with higher vs lower risk scores have not been clearly demonstrated.

To compare the optimal levels of treated BP between high- and low-risk patients, this study assessed the cardiorenal event rate and all-cause mortality according to achieved BP and baseline risk status in population-based cohorts of Korean adults from the National Health Information Database (NHID).

## Methods

### Participants

This cohort study was conducted using the NHID, which is a public database for the entire population of Korea maintained by the National Health Insurance Service (NHIS). The NHID consists of data collected from 2002 onward and includes demographic characteristics, nationwide health screenings, and NHIS reimbursement record information (eMethods 1 in the Supplement). The study was approved by the institutional review board of Kangwon National University Hospital. The need for informed consent was waived because the data used were deidentified prior to analysis. The study followed the Strengthening the Reporting of Observational Studies in Epidemiology (STROBE) reporting guideline for cohort studies.

The NHIS constructed a sample cohort from the NHID and released it for research purposes (eMethods 2 in the Supplement). From among 5.15 million adults aged 40 to 79 years who underwent health screening in 2002 or 2003, 514 866 participants were randomly selected. They underwent biennial health screenings thereafter and were followed up through December 31, 2015. In this study, 985 participants with insufficient data to assess baseline risk status, 17 185 who reported a medical history of stroke or heart disease or were diagnosed with chronic kidney disease, and 9284 who died or experienced a critical cardiorenal event before baseline (ie, January 1, 2006) were excluded to avoid confounding of the outcome risk by preexisting conditions. The final analysis of the primary cohort included 487 412 participants (eFigure 1A in the Supplement).

Additionally, a secondary cohort was constructed from the NHID to assess baseline risk status using the ACC/AHA (or Framingham) risk score, a popular scale for predicting cardiovascular risk. One million participants were randomly selected from 7.13 million adults aged 40 to 79 years who underwent health screening in 2009 when the measurement of high-density lipoprotein cholesterol (a component of the ACC/AHA or Framingham score) began. Biennial health screening data and NHIS reimbursement records were collected from 2006 to 2017. Among the 1 million participants, 21 543 with insufficient data, 54 220 with a history of stroke or heart disease or with an estimated glomerular filtration rate of less than 30 mL/min/1.73 m^2^, and 8674 who died or experienced a critical cardiorenal event before baseline (ie, January 1, 2010) were excluded from the study. The final analysis of the secondary cohort included 915 563 participants (eFigure 1B in the Supplement).

### Blood Pressure and Covariates

During health screenings, BP was measured using sphygmomanometers or oscillometric devices after a 5-minute rest, with repeated measurements using sphygmomanometers performed after 2 minutes when the BP was 140/90 mm Hg or greater. The treated and untreated BP records were collected separately, as BP thresholds for adverse outcomes were shown to be substantially different between actively treated and untreated patients.^6^ Treated BP was defined as having had an antihypertensive medication prescribed for 90 days or more in the year of BP measurement, while untreated was defined as having received an antihypertensive prescription for less than 90 days in that year (eMethods 3 in the Supplement).

In each year of follow-up, mean BP values, insurance contribution, alcohol consumption, exercise frequency, and body mass index were calculated from the year of registration (ie, from 2002 and 2006 in the primary and secondary cohorts, respectively) (eTable 1 in the Supplement) to incorporate changes in the variables over time. Using the yearly updated mean values, I categorized variables as follows: systolic BP (<110, 110-119, 120-129, 130-139, 140-149, 150-159, or ≥160 mm Hg), income level (high, middle, or low), alcohol consumption (0, 0.1-0.4, 0.5-1.4, 1.5-2.9, or ≥3.0 drinks per day), exercise frequency (<1, 1-2, 3-4, or ≥5 days per week), and body mass index (<18.5, 18.5-22.9, 23.0-24.9, 25.0-29.9, or ≥30.0 [calculated as weight in kilograms divided by height in meters squared]). In each year, I also determined the status of diabetes (yes or no), hyperlipidemia (yes or no), proteinuria (yes or no), and smoking (never, former, or active smoker). Using baseline data, I categorized age (40-44, 45-49, 50-54, 55-59, 60-64, 65-69, 70-74, or 75-79 years), sex (male or female), and a family history of cardiovascular disease (yes or no). Categories for missing values were included for all variables to minimize the loss of cases during analysis.

### Risk Categories and Outcomes

Five risk factors (hypertension, diabetes, hyperlipidemia, proteinuria, and active smoking) were identified using the health screening and NHIS reimbursement records (eTable 2 in the Supplement): hypertension was defined as BP 140/90 mm Hg or greater or prescription of antihypertensives for 90 days or more per year; diabetes as fasting glucose level of 126 mg/dL or greater (to convert to millimoles per liter, multiply by 0.0555) or a prescription of antidiabetic medication for 90 or more days per year; hyperlipidemia as total cholesterol level of 240 mg/dL or greater (to convert to millimoles per liter, multiply by 0.0259) or prescription of statins for 90 or more days per year; proteinuria as urine dipstick albumin 1+ or greater once or greater than trace amount at least twice; and active smoking as current smoking. Participants in both cohorts were grouped into 3 risk categories by the number of risk factors present at baseline (≥3, 2, or ≤1) and additionally by the risk scores from cardiovascular risk calculators (eMethods 4 in the Supplement). The primary cohort participants were categorized using the Systematic Coronary Risk Evaluation (SCORE) system for low-risk regions (≥7.5%, 2.5%-7.4%, or <2.5%),^7^ and the secondary cohort participants were categorized using a Korean prediction model (≥15%, 7.5%-14%, or <7.5%)^8^ that was developed on the basis of the 2013 ACC/AHA calculator^9^ owing to concerns about overestimation of risk when using the Framingham or original ACC/AHA score in the Korean population.^8,10^

The study outcomes were identified through December 31, 2015, in the primary cohort and through December 31, 2017, in the secondary cohort. Critical cardiorenal events were identified as a composite of admission to the critical care unit with cardiovascular or chronic kidney disease, revascularization for myocardial infarction or stroke, and newly developed end-stage kidney disease using in-hospital procedures and primary medical diagnosis in NHIS reimbursement record information (eMethods 5 and eTable 3 in the Supplement). All-cause deaths were confirmed using death records that were included in the NHID.

### Statistical Analysis

Statistical analyses were conducted using SAS statistical software version 9.4 (SAS Institute). The outcomes of interest were a critical cardiorenal event and all-cause death; the primary exposure of interest was treated systolic BP. Multivariable-adjusted hazard ratios (HRs) were estimated using Cox models with time-varying covariates (as time-lagged covariates)^11^ (eTable 1 in the Supplement). In all models, the treated and untreated systolic BP levels were both entered as time-varying covariates to obtain HRs for treated BP adjusted for untreated state levels. The yearly updated levels of income, body mass index, exercise frequency, alcohol consumption, and the yearly determined status of diabetes, hyperlipidemia, proteinuria, and smoking were also included as time-varying covariates. Baseline age, sex, and family history of cardiovascular disease were entered as fixed covariates. Cox models with time-varying covariates of diastolic BP replacing systolic BP were also constructed. The Cox analyses were stratified according to baseline risk categories to compare the risk estimates across the risk categories. The summary effects with 95% confidence intervals of the primary and secondary cohorts were calculated using the DerSimonian-Laird random-effects model.^12^

To estimate absolute risk based on the event rates in the reference group, age-standardized annual event rates and 95% confidence intervals were calculated by multiplying the HRs and 95% confidence intervals by the mean of the age-specific annual rates in the reference group (ie, a treated systolic BP of 120-129 mm Hg). Age-specific annual event rates were calculated by dividing the number of events by the number of person-years in each age category, except for extremes of age categories, within the reference group. In addition to subgroup analyses stratified according to age (≥65 or <65 years) or sex, several sensitivity analyses were conducted: first, by restricting to participants who initiated antihypertensive treatment prior to baseline or by excluding participants who were treated in the year of registration; second, by further adjusting for antihypertensive compliance (regular, irregular, or never use) (eMethods 3 in the Supplement) to explore the effect of adherence on the risk estimates; third, by counting risk factors after exclusion of proteinuria, which had not been regarded as a major risk factor; and fourth, by using the World Health Organization/International Society of Hypertension (WHO/ISH) or Framingham risk scores.^13,14^ Data are presented as numbers and percentages, means and standard deviations, medians and interquartile ranges, HRs and 95% confidence intervals, or annual event rates and 95% confidence intervals.

## Results

### Baseline Characteristics

Table 1 shows the baseline characteristics of the study participants according to the presence of risk factors. The primary cohort included 487 412 participants (263 385 [54.0%] male; median [interquartile range] age, 50 [44-59] years). The secondary cohort included 915 563 participants (459 053 [50.1%] male; median [interquartile range] age, 52 [46-60] years). Compared with participants with 1 or 0 risk factors, participants with multiple risk factors were older and included greater proportions of participants who were male, were overweight, and consumed alcohol. eTable 4 in the Supplement shows the characteristics according to the risk scores from cardiovascular risk calculators. Participants with higher scores were much older than those with lower scores.

**Table 1.** Baseline Characteristics of Study Participants According to Risk Categories^a^

Characteristic	Primary Cohort			Secondary Cohort		
	≤1 Risk Factor	2 Risk Factors	≥3 Risk Factors	≤1 Risk Factor	2 Risk Factors	≥3 Risk Factors
No. of participants	337 796	110 798	38 818	649 376	189 493	76 694
Blood pressure, mean (SD), mm Hg						
Systolic blood pressure	123.0 (15.0)	134.7 (15.1)	137.4 (14.7)	121.1 (12.8)	131.1 (13.6)	133.4 (13.4)
Diastolic blood pressure	77.0 (9.7)	84.2 (9.5)	85.7 (9.3)	75.5 (8.5)	81.5 (8.9)	82.5 (8.9)
Age, median (IQR), y^b^	49 (44-58)	53 (46-61)	53 (46-61)	50 (44-58)	56 (49-64)	56 (49-64)
Men, No. (%)	160** **420 (47.5)	73** **103 (66.0)	29** **862 (76.9)	287** **978 (44.3)	116** **376 (61.4)	54** **699 (71.3)
Family history of cardiovascular disease, No. (%)	33** **573 (9.9)	11** **919 (10.8)	4142 (10.7)	57** **114 (8.8)	17** **504 (9.2)	6855 (8.9)
Income level, No. (%)						
High	131** **651 (39.0)	39** **807 (35.9)	13** **348 (34.4)	150** **910 (23.2)	41** **373 (21.8)	15** **850 (20.7)
Middle	133** **247 (39.4)	44** **617 (40.3)	15** **757 (40.6)	242** **956 (37.4)	71** **790 (37.9)	29** **053 (37.9)
Low	72** **898 (21.6)	26** **374 (23.8)	9713 (25.0)	253** **750 (39.1)	75** **828 (40.0)	31** **613 (41.2)
Unknown	0	0	0	1760 (0.3)	502 (0.3)	178 (0.2)
Hypertension, No. (%)	110** **883 (32.8)	94** **127 (85.0)	36** **885 (95.0)	147** **976 (22.8)	147** **591 (77.9)	70** **941 (92.5)
Diabetes, No. (%)	9824 (2.9)	27** **090 (24.4)	25** **904 (66.7)	18** **024 (2.8)	46** **907 (24.8)	53** **715 (70.0)
Hyperlipidemia, No. (%)	29** **087 (8.6)	51** **029 (46.1)	28** **639 (73.8)	70** **932 (10.9)	103** **615 (54.7)	60** **891 (79.4)
Proteinuria, No. (%)	2681 (0.8)	5543 (5.0)	7226 (18.6)	7960 (1.2)	11** **887 (6.3)	17** **841 (23.3)
Smoking, No. (%)						
Never smoked	260** **299 (77.1)	57** **585 (52.0)	12** **864 (33.1)	458** **313 (70.6)	94** **105 (49.7)	27** **173 (35.4)
Former smoker	33** **240 (9.8)	8180 (7.4)	1951 (5.0)	104** **795 (16.1)	26** **182 (13.8)	8471 (11.0)
Current smoker	37** **875 (11.2)	43** **807 (39.5)	23** **743 (61.2)	84** **860 (13.1)	68** **986 (36.4)	40** **974 (53.4)
Unknown	6382 (1.9)	1226 (1.1)	260 (0.7)	1408 (0.2)	220 (0.1)	76 (0.1)
BMI, No. (%)						
<16.0	7932 (2.3)	1787 (1.6)	415 (1.1)	14** **906 (2.3)	2407 (1.3)	651 (0.8)
16.0-22.9	132** **940 (39.4)	31** **591 (28.5)	9323 (24.0)	266** **653 (41.1)	51** **364 (27.1)	16** **378 (21.4)
23.0-24.9	94** **325 (27.9)	31** **120 (28.1)	10** **684 (27.5)	176** **986 (27.3)	52** **477 (27.7)	20** **113 (26.2)
25.0-29.9	95** **865 (28.4)	42** **198 (38.1)	16** **505 (42.5)	177** **043 (27.3)	74** **820 (39.5)	34** **404 (44.9)
≥30.0	6609 (2.0)	4071 (3.7)	1883 (4.9)	13** **760 (2.1)	8408 (4.4)	5140 (6.7)
Unknown	125 (0.0)	31 (0.0)	8 (0.0)	28 (0.0)	17 (0.0)	8 (0.0)
Physical exercise, No. (%)						
<1 d/wk	156** **624 (46.4)	48** **153 (43.5)	15** **552 (40.1)	321** **981 (49.6)	93** **639 (49.4)	37** **335 (48.7)
1-2 d/wk	101** **879 (30.2)	35** **819 (32.3)	13** **613 (35.1)	160** **713 (24.7)	45** **434 (24.0)	18** **581 (24.2)
3-4 d/wk	50** **017 (14.8)	17** **618 (15.9)	6531 (16.8)	92** **969 (14.3)	26** **970 (14.2)	11** **091 (14.5)
≥5 d/wk	24** **541 (7.3)	8264 (7.5)	2928 (7.5)	67** **213 (10.4)	21** **738 (11.5)	9029 (11.8)
Unknown	4735 (1.4)	944 (0.9)	194 (0.5)	6500 (1.0)	1712 (0.9)	658 (0.9)
Alcohol consumption, No. (%)						
<0.1 Drinks/d	200** **901 (59.5)	50** **691 (45.8)	14** **228 (36.7)	388** **416 (59.8)	96** **767 (51.1)	34** **394 (44.8)
0.1-0.4 Drinks/d	47** **840 (14.2)	14** **018 (12.7)	4540 (11.7)	59** **631 (9.2)	12** **726 (6.7)	4640 (6.1)
0.5-1.4 Drinks/d	35** **754 (10.6)	16** **032 (14.5)	6537 (16.8)	88** **777 (13.7)	28** **817 (15.2)	12** **377 (16.1)
1.5-2.9 Drinks/d	27** **575 (8.2)	15** **157 (13.7)	6671 (17.2)	46** **706 (7.2)	20** **485 (10.8)	9851 (12.8)
≥3.0 Drinks/d	21** **870 (6.5)	14** **090 (12.7)	6658 (17.2)	51** **668 (8.0)	26** **948 (14.2)	13** **936 (18.2)
Unknown	3856 (1.1)	810 (0.7)	184 (0.5)	14** **178 (2.2)	3750 (2.0)	1496 (2.0)

Abbreviations: BMI, body mass index (calculated as weight in kilograms divided by height in meters squared); IQR, interquartile range.

^a^ The risk categories were grouped by the number of risk factors present at baseline (on January 1, 2006, and January 1, 2010, in the primary and secondary cohorts, respectively), ie, 3 or more, 2, and 1 or fewer of the 5 risk factors (hypertension, diabetes, hyperlipidemia, proteinuria, and smoking).

^b^ The values are the ages at the end of 2005 in the primary cohort and at the end of 2009 in the secondary cohort.

### Cardiorenal Risk and Mortality

During study periods, 225 103 participants in the primary cohort and 360 503 in the secondary cohort were actively treated with antihypertensive drugs for at least 1 year. Over the 10 and 8 years of follow-up, 23 421 participants in the primary cohort experienced critical cardiorenal events and 36 373 died. In the secondary cohort, 27 871 participants experienced critical cardiorenal events and 36 127 died. In the 2 cohorts combined, 28 411 of 51 292 cardiorenal incidents and 33 102 of 72 500 deaths were noted in participants ever treated with antihypertensive medications (eTable 5 in the Supplement).

**Figure 1.**
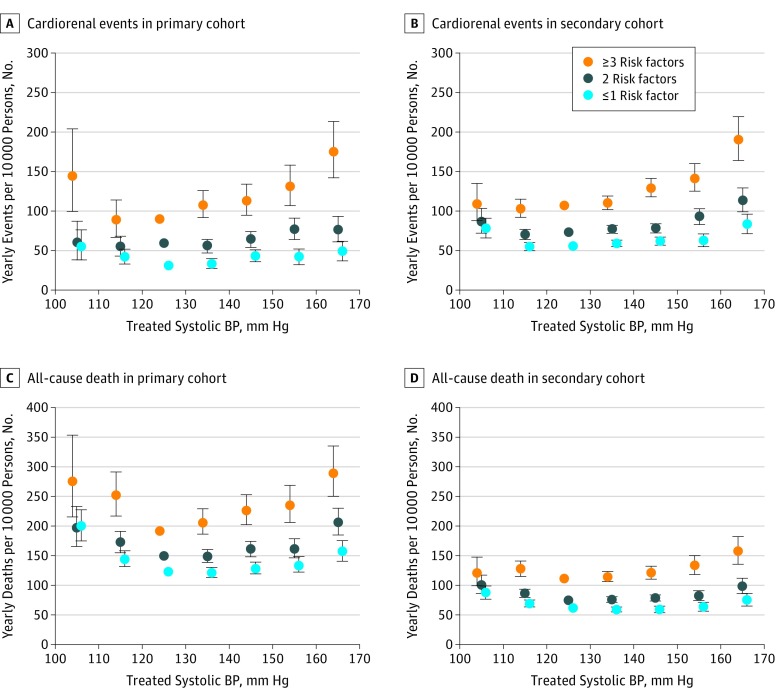
Yearly Event Rates According to Treated Systolic Blood Pressure (BP) and Risk Categories One-year rates were estimated in the primary (A and C) and secondary (B and D) cohorts by multiplying the hazard ratios by the mean of the age-specific rates in the reference group. The time-averaged systolic BP of 120 to 129 mm Hg served as the reference. All analyses were adjusted for age, sex, family history of cardiovascular disease, income level, smoking, alcohol consumption, exercise frequency, body mass index, diabetes, hyperlipidemia, and proteinuria. The cardiorenal event was a composite of admission to the critical care unit with cardiovascular or chronic kidney disease, revascularization for myocardial infarction or stroke, and new-onset end-stage kidney disease. Error bars indicate 95% confidence intervals.

For critical cardiorenal events, the absolute risk increase associated with inadequately treated BP was greater in participants with multiple vs 1 or 0 risk factors (Figure 1A and B; eTable 6 and eTable 7 in the Supplement), and the HRs for cardiorenal event increased significantly as the treated systolic BP increased to more than 130 to 140 mm Hg (Table 2). With all-cause mortality, the absolute risk of inadequately treated BP was also greater in participants with more risk factors (Figure 1C and D). The HR for all-cause mortality for patients with 3 or more risk factors and treated systolic BP within the range of 110 to 119 mm Hg was 1.21 (95% CI, 1.07-1.37); 130 to 139 mm Hg, 1.04 (95% CI, 0.98-1.11); 140 to 149 mm Hg, 1.12 (95% CI, 1.05-1.20); 150 to 159 mm Hg, 1.21 (95% CI, 1.11-1.32); and 160 mm Hg or greater, 1.46 (95% CI, 1.32-1.62) compared with high-risk patients with BP of 120 to 129 mm Hg. For participants with 1 or 0 risk factors and treated systolic BP within the range of 110 to 119 mm Hg, the hazard ratio was 1.14 (95% CI, 1.07-1.22); 130 to 139 mm Hg, 0.97 (95% CI, 0.93-1.02); 140 to 149 mm Hg, 1.00 (95% CI, 0.91-1.09); 150 to 159 mm Hg, 1.06 (95% CI, 0.99-1.14); and 160 mm Hg or greater, 1.26 (95% CI, 1.15-1.37) (Table 2).

**Table 2.** Hazard Ratios According to Treated Systolic BP and Risk Categories^a^

Outcome	Hazard Ratio (95% CI)						
	Treated Systolic BP, mm Hg						
	<110	110-119	120-129^b^	130-139	140-149	150-159	≥160
Critical cardiorenal event^c^							
≥3 Risk factors	1.18 (0.87-1.59)	0.97 (0.88-1.06)	1 [Reference]	1.07 (0.98-1.17)	1.19 (1.11-1.28)	1.31 (1.19-1.43)	1.69 (1.52-1.88)
2 Risk factors	1.11 (0.96-1.28)	0.96 (0.89-1.03)	1 [Reference]	1.01 (0.93-1.10)	1.06 (1.00-1.12)	1.21 (1.11-1.32)	1.33 (1.00-1.77)
≤1 Risk factor	1.35 (1.20-1.53)	1.06 (0.91-1.22)	1 [Reference]	1.04 (0.99-1.10)	1.13 (1.07-1.20)	1.13 (1.04-1.23)	1.34 (1.11-1.62)
All-cause mortality							
≥3 Risk factors	1.24 (0.95-1.61)	1.21 (1.07-1.37)	1 [Reference]	1.04 (0.98-1.11)	1.12 (1.05-1.20)	1.21 (1.11-1.32)	1.46 (1.32-1.62)
2 Risk factors	1.34 (1.19-1.50)	1.16 (1.08-1.24)	1 [Reference]	1.01 (0.97-1.06)	1.07 (1.01-1.13)	1.09 (1.02-1.18)	1.36 (1.25-1.48)
≤1 Risk factor	1.51 (1.32-1.74)	1.14 (1.07-1.22)	1 [Reference]	0.97 (0.93-1.02)	1.00 (0.91-1.09)	1.06 (0.99-1.14)	1.26 (1.15-1.37)

Abbreviation: BP, blood pressure.

^a^ Hazard ratios were estimated in each of the primary and secondary cohorts using Cox models with time-varying covariates. Then, the summary effects and 95% confidence intervals of the 2 cohorts were calculated using the DerSimonian-Laird random-effects model. All analyses were adjusted for age, sex, family history of cardiovascular disease, income level, smoking, alcohol consumption, exercise frequency, body mass index, diabetes, hyperlipidemia, and proteinuria.

^b^ The time-averaged systolic BP of 120 to 129 mm Hg served as the reference.

^c^ The critical cardiorenal event was a composite of admission to the critical care unit with cardiovascular or chronic kidney disease, revascularization for myocardial infarction or stroke, and new-onset end-stage kidney disease.

**Figure 2.**
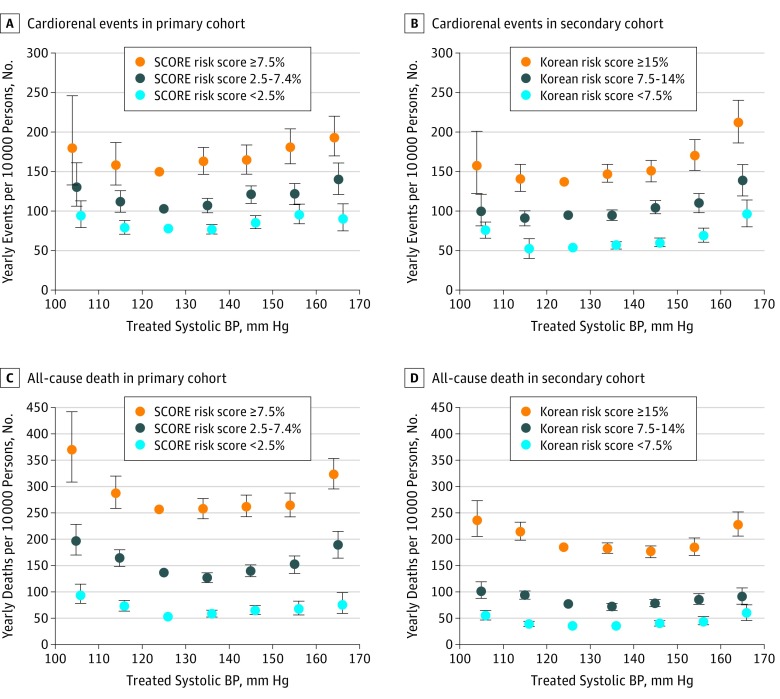
Yearly Event Rates According to Risk Scores From Systematic Coronary Risk Evaluation (SCORE) or Korean Prediction Model One-year rates were estimated in the primary (A and C) and secondary (B and D) cohorts by multiplying the hazard ratios by the mean of the age-specific rates in the reference group (systolic blood pressure [BP], 120-129 mm Hg). All analyses were adjusted for age, sex, family history of cardiovascular disease, income level, smoking, alcohol consumption, exercise frequency, body mass index, diabetes, hyperlipidemia, and proteinuria. The cardiorenal event was a composite of admission to the critical care unit with cardiovascular or chronic kidney disease, revascularization for myocardial infarction or stroke, and new-onset end-stage kidney disease. Error bars indicate 95% confidence intervals.

However, when categorized according to the scores calculated from the SCORE or Korean prediction model, the risk thresholds of treated BP were not left shifted in participants with higher risk scores (Figure 2; eTable 8 in the Supplement). In age-stratified analysis, the absolute risk of both high and low BP was greater in elderly (≥65 years) vs middle aged (<65 years) participants, and the mortality threshold of treated BP was slightly right shifted in elderly participants (eFigure 2 and eTable 9 in the Supplement). In sex-stratified analysis, the absolute risk of high BP was slightly greater in male vs female participants, while the mortality threshold of treated BP was slightly left shifted in male participants. The risk thresholds of treated diastolic BP are shown in eFigure 3 in the Supplement, and those of untreated BP are shown in eFigure 4 in the Supplement.

### Sensitivity Analysis

When the analyses were restricted to prevalent users of antihypertensive medications or even when past users who were treated in the year of registration were excluded, the mortality thresholds of treated BP were also left shifted in participants with multiple risk factors (eFigure 5 and eTable 10 in the Supplement). When further adjusted for drug compliance, the risk estimates were similar to those of the primary analysis (eFigure 6 and eTable 11 in the Supplement). Even when counting the risk factors after exclusion of proteinuria, the main results persisted with no substantial change (eFigure 7 and eTable 12 in the Supplement). However, when categorized according to the WHO/ISH or Framingham score, there was no consistent trend in the risk thresholds of treated BP across the score categories (eFigure 8 and eTable 8 in the Supplement).

## Discussion

In this cohort study of Korean adults who had no known cardiorenal disease, the absolute increase in cardiorenal and mortality risk associated with inadequately treated BP was greater in participants with multiple vs 1 or 0 risk factors. While the cardiorenal risk increased as the treated systolic BP increased to more than 130 to 140 mm Hg, the mortality thresholds of treated systolic BP (ie, the thresholds above which all-cause mortality increased with statistical significance) in participants with 3 or more, 2, and 1 or 0 risk factors were 130 to 140 mm Hg, 140 to 150 mm Hg, and 150 to 160 mm Hg, respectively. However, when categorized according to the scores from cardiovascular risk calculators, there was no consistent trend in risk thresholds of treated BP across the risk score categories. To my knowledge, this is the first study clearly demonstrating that the mortality threshold of treated BP is associated with different levels of risk status. This study, however, failed to discriminate the risk groups according to different BP thresholds when grouped using risk calculators.

Previously, our research group conducted a cohort study to compare BP-associated risk between users and nonusers of antihypertensive drugs. In that study,^6^ J-shaped associations between achieved BP and adverse outcomes were observed among active users, suggesting the lowering of BP to normal or below normal levels is not beneficial or may even be harmful in actively treated patients. However, recent meta-analyses^15,16^ of clinical trials have reported that intensive lowering of systolic BP to below 130 mm Hg has beneficial effects on cardiovascular and mortality outcomes. These conflicting findings raised a possibility that some but not all patients may benefit from intensive BP lowering and indicated that further research was needed. Contrary to the previous analyses, the present study compared the optimal BP between higher- and lower-risk patients and found that optimally treated BP having the most favorable outcome was left shifted in patients with multiple vs 1 or 0 risk factors. This suggests that BP targets should be individualized and that intensive BP control should be considered for higher- rather than lower-risk patients. This finding can also partially explain the discordance between trials with different patient characteristics.^17,18,19^

Notably, the present study found that the J-curve of treated BP for all-cause mortality was apparent and right shifted in low-risk patients. To date, the J-curve of BP has been observed mostly in post hoc analyses of clinical trials or cohort studies that involved high-risk patients,^20,21,22^ while most of the cohort studies that included low-risk individuals have observed a linear association between BP and outcome risk.^23^ The previous observations appear inconsistent with the present findings. However, the linear associations observed in cohort studies that did not account for antihypertensive use might be derived from untreated BP rather than actively treated BP as shown in this (eFigure 4 in the Supplement) and another study.^6^ The finding of right-shifted J-curve in low-risk patients could be explained after a search of the literature. Several meta-analyses^1,2^ of clinical trials reported that intensive BP lowering had clear benefits for cardiovascular outcomes rather than for all-cause mortality, and the cardiovascular benefits from BP lowering might in fact be larger in higher-risk patients, as reaffirmed by this study. On the other hand, intensive BP lowering might increase the risk of treatment-related adverse events, including syncope, injurious falls, electrolyte disturbance, bradycardia, and acute kidney injury,^17,18,19,24,25^ and, potentially, the risk for a coronary event due to impaired coronary perfusion.^6,20,21^ As BP decreases progressively, the adverse effects might increase progressively and outweigh the cardiorenal benefits when below a certain BP threshold. This results in increased all-cause mortality as a manifestation of the overall effects of lowered BP. Accordingly, the smaller cardiorenal benefits for lower-risk patients would incur the mortality threshold of treated BP to be right shifted in lower- vs higher-risk patients.

When participants were categorized according to the scores from the SCORE and Korean models (or the WHO/ISH and Framingham risk scores), the optimal level of treated BP was not lower in participants with higher- vs lower-risk scores. This suggests that current risk calculators are less valid among Korean or Asian populations, so there is a need for more generalized validated risk assessment tools for determining optimal BP targets. This is a point for future research; the finding should be confirmed in other populations. The risk calculators, which include age as a prediction factor, calculate the risk score to be higher in older individuals. However, the mortality threshold of treated BP was slightly right shifted rather than left shifted in elderly participants in the present cohorts. The benefits of intensive BP lowering might be offset by the treatment-related adverse effects, which could be of larger effect in elderly individuals.^24,25,26^ In addition to age, the level to which BP is treated or untreated is included as a predictor in most risk calculators. Consequently, the risk score is calculated to be higher in patients with poorly controlled BP or with very high BP. Earlier initiation of proper BP management is reasonable in such patients. However, lowering BP below standard targets is not an appropriate treatment strategy and may even be harmful in patients with intractable or very high BP, as shown in post hoc analyses of the Systolic Blood Pressure Intervention Trial.^27,28^ Old age itself, high baseline BP, or poor response to treatment may not be a good indicator for intensive BP control, although they are very helpful predictors of future cardiovascular events and may help therapeutic decisions for certain conditions.

### Limitations

This study has several limitations to consider. First, it might be difficult to set precise BP targets considering the observational nature of the study. Additionally, the approach to BP measurements should be considered because BP obtained from routine office measurement is generally higher than that obtained via ambulatory or home measurement.^29,30^ However, even when using ambulatory or home BP, the risk thresholds of treated BP would be left shifted in higher- vs lower-risk patients. Second, there were missing data on treated (or untreated) BP because this study was not a planned intervention study (eTable 5 in the Supplement). In addition, some participants did not undergo health screenings regularly despite the biennial screening recommendation (eTable 13 in the Supplement). Nonetheless, when the analysis restricted to prevalent antihypertensive users who initiated treatment prior to baseline, the main results persisted while missing data on treated BP were minimized. In addition, caution is required in applying the findings to populations with serious conditions or other ethnicity because the study involved participants who had no known cardiorenal disease and who resided in Korea.

## Conclusions

This population-based study in Korea supports the need for individualized management of BP considering more intensive control in patients at higher risk. However, modification may be required for current risk calculators to determine the optimal target BP.

## Notes

Supplement. **eMethods 1.** National Health Information Database (NHID) **eMethods 2.** Cohort Participants **eMethods 3.** Covariates **eMethods 4.** Risk Categories **eMethods 5.** Outcomes **eFigure 1.** Flow Charts of Participant Selection in the Primary (A) and Secondary (B) Cohorts **eFigure 2.** Yearly Event Rates in Age- or Sex-Stratified Subgroups **eFigure 3.** Yearly Event Rates According to Treated Diastolic BP and Risk Categories **eFigure 4.** Yearly Event Rates According to Untreated Systolic BP and Risk Categories **eFigure 5.** Yearly Event Rates in Prevalent (A and C) or Recent (B and D) Antihypertensive Users **eFigure 6.** Yearly Event Rates After Further Adjustment for Compliance **eFigure 7.** Yearly Event Rates in Risk Categories Grouped by Risk Factors After Exclusion of Proteinuria **eFigure 8.** Yearly Event Rates According to WHO/ISH or Framingham Scores **eTable 1.** Time-Lagged Covariates for Subsequent Years **eTable 2.** NHIS Generic Name Codes for Antidiabetics, Antihypertensives, and Statins **eTable 3.** NHIS Codes for Arterial Revascularization Procedures and Peritoneal Dialysates **eTable 4.** Baseline Characteristics of the Study Participants According to Risk Scores From Cardiovascular Risk Calculators **eTable 5.** Cardiorenal Event and All-Cause Death According to Systolic BP and Risk Categories **eTable 6.** Yearly Event Rates According to Achieved BP and Risk Categories in the Primary Cohort **eTable 7.** Yearly Event Rates According to Achieved BP and Risk Categories in the Secondary Cohort **eTable 8.** Yearly Event Rates According to Risk Scores From Cardiovascular Risk Calculators **eTable 9.** Yearly Event Rates in Age- or Sex-Stratified Subgroups **eTable 10.** Yearly Event Rates in Prevalent or Recent Antihypertensive Users **eTable 11.** Yearly Event Rates After Further Adjustment for Compliance **eTable 12.** Yearly Event Rates in Risk Categories Grouped by Risk Factors After Exclusion of Proteinuria **eTable 13.** Participation Rates in Health Screenings During the Study Periods **eReferences**
