# Supplementary material for: Association of Optimal Blood Pressure With Critical Cardiorenal Events and Mortality in High-Risk and Low-Risk Patients Treated With Antihypertension Medications
Source: JAMA Netw Open. 2019 Aug 23;2(8):e199307. doi: 10.1001/jamanetworkopen.2019.9307 (PMC6714012; doi:10.1001/jamanetworkopen.2019.9307)
Supplement: Supplement. — eMethods 1. National Health Information Database (NHID) eMethods 2. Cohort Participants eMethods 3. Covariates eMethods 4. Risk Categories eMethods 5. Outcomes eFigure 1. Flow Charts of Participant Selection in the Primary (A) and Secondary (B) Cohorts eFigure 2. Yearly Event Rates in Age- or Sex-Stratified Subgroups eFigure 3. Yearly Event Rates According to Treated Diastolic BP and Risk Categories eFigure 4. Yearly Event Rates According to Untreated Systolic BP and Risk Categories eFigure 5. Yearly Event Rates in Prevalent (A and C) or Recent (B and D) Antihypertensive Users eFigure 6. Yearly Event Rates After Further Adjustment for Compliance eFigure 7. Yearly Event Rates in Risk Categories Grouped by Risk Factors After Exclusion of Proteinuria eFigure 8. Yearly Event Rates According to WHO/ISH or Framingham Scores eTable 1. Time-Lagged Covariates for Subsequent Years eTable 2. NHIS Generic Name Codes for Antidiabetics, Antihypertensives, and Statins eTable 3. NHIS Codes for Arterial Revascularization Procedures and Peritoneal Dialysates eTable 4. Baseline Characteristics of the Study Participants According to Risk Scores From Cardiovascular Risk Calculators eTable 5. Cardiorenal Event and All-Cause Death According to Systolic BP and Risk Categories eTable 6. Yearly Event Rates According to Achieved BP and Risk Categories in the Primary Cohort eTable 7. Yearly Event Rates According to Achieved BP and Risk Categories in the Secondary Cohort eTable 8. Yearly Event Rates According to Risk Scores From Cardiovascular Risk Calculators eTable 9. Yearly Event Rates in Age- or Sex-Stratified Subgroups eTable 10. Yearly Event Rates in Prevalent or Recent Antihypertensive Users eTable 11. Yearly Event Rates After Further Adjustment for Compliance eTable 12. Yearly Event Rates in Risk Categories Grouped by Risk Factors After Exclusion of Proteinuria eTable 13. Participation Rates in Health Screenings During the Study Periods eReferences [file jamanetwopen-2-e199307-s001.pdf]

Supplementary Online Content

Jung HH. Association of optimal blood pressure with critical cardiorenal events and mortality in high-risk and low-risk patients treated with antihypertension medications. *JAMA Netw Open*. 2019;2(8):e199307. doi:10.1001/jamanetworkopen.2019.9307

eMethods 1. National Health Information Database (NHID)
eMethods 2. Cohort Participants
eMethods 3. Covariates
eMethods 4. Risk Categories
eMethods 5. Outcomes
eFigure 1. Flow Charts of Participant Selection in the Primary (A) and Secondary (B) Cohorts
eFigure 2. Yearly Event Rates in Age- or Sex-Stratified Subgroups
eFigure 3. Yearly Event Rates According to Treated Diastolic BP and Risk Categories
eFigure 4. Yearly Event Rates According to Untreated Systolic BP and Risk Categories
eFigure 5. Yearly Event Rates in Prevalent (A and C) or Recent (B and D) Antihypertensive Users
eFigure 6. Yearly Event Rates After Further Adjustment for Compliance
eFigure 7. Yearly Event Rates in Risk Categories Grouped by Risk Factors After Exclusion of Proteinuria
eFigure 8. Yearly Event Rates According to WHO/ISH or Framingham Scores
eTable 1. Time-Lagged Covariates for Subsequent Years
eTable 2. NHIS Generic Name Codes for Antidiabetics, Antihypertensives, and Statins
eTable 3. NHIS Codes for Arterial Revascularization Procedures and Peritoneal Dialysates
eTable 4. Baseline Characteristics of the Study Participants According to Risk Scores From Cardiovascular Risk Calculators
eTable 5. Cardiorenal Event and All-Cause Death According to Systolic BP and Risk Categories
eTable 6. Yearly Event Rates According to Achieved BP and Risk Categories in the Primary Cohort
eTable 7. Yearly Event Rates According to Achieved BP and Risk Categories in the Secondary Cohort
eTable 8. Yearly Event Rates According to Risk Scores From Cardiovascular Risk Calculators
eTable 9. Yearly Event Rates in Age- or Sex-Stratified Subgroups
eTable 10. Yearly Event Rates in Prevalent or Recent Antihypertensive Users
eTable 11. Yearly Event Rates After Further Adjustment for Compliance
eTable 12. Yearly Event Rates in Risk Categories Grouped by Risk Factors After Exclusion of Proteinuria
eTable 13. Participation Rates in Health Screenings During the Study Periods
eReferences

This supplementary material has been provided by the authors to give readers additional information about their work.

© 2019 Jung HH. *JAMA Network Open.*

eMethods 1. National Health Information Database (NHID)

The NHID, a public database for the whole population of South Korea, was established and is being maintained by the National Health Insurance Service (NHIS). Details on the NHID are published elsewhere.[1,2]

The NHID covers data from 2002 onwards, and comprises the following.
(a) eligibility data: demographics, income based insurance contributions, and date of death
(b) health screening records: medical history, health behavior, physical exam, and laboratory exam
(c) reimbursement records of the NHIS: prescribed drugs, medical procedures, outpatient visits, hospitalizations, and lists of medical diagnosis
(d) health care provider data: medical institutions, equipment, and human resources

The data in each resource was assembled using de-identified join keys, which replace personal identification numbers assigned to citizens of Korea.

Nationwide health screenings are performed for citizens aged ≥40 years, generally at 2-year intervals, in hospitals or medical centers. During the health screening, information on medical history and health behaviors are obtained using standardized questionnaires. Trained medical staff perform physical examinations including blood pressure (BP) measurement. Blood and urine samples are obtained after at least an 8-h fast.

Serum creatinine and high-density lipoprotein (HDL) and low-density lipoprotein (LDL) cholesterols have been measured from 2009 health screening.

© 2019 Jung HH. *JAMA Network Open.*

eMethods 2. Cohort Participants

Two cohorts of Korean adults aged ≥40 years, who had no known cardiorenal disease, were constructed from the NHID (eFigure 1).

A. Primary cohort

The NHIS constructed a sample cohort from the NHID.[2,3] A total of 514,866 participants were randomly selected from 5.15 million adults aged 40–79 years at the end of 2002, who had undergone a health screening in 2002–2003. The NHIS collected health information data from 2002 through 2015.

The primary cohort participants were selected according to the following criteria.
(a) Inclusion: men and women with available information to assess risk status at baseline (on January 1, 2006)
(b) Exclusion: participants who reported having heart disease or stroke during health screenings between 2002 and 2005, those who were diagnosed with chronic kidney disease (ICD-10, N18) between 2002 and 2005, and those who died or in whom a critical cardiovascular event occurred before January 1, 2006

B. Secondary cohort

I constructed a secondary cohort from the NHID. One million participants were randomly selected from 7.13 million adults aged 40–79 years in 2009, who had undergone a health screening in 2009. I collected health information data from 2006 through 2017.

The secondary cohort participants were selected according to the following criteria.
(a) Inclusion: men and women with available information to assess risk status at baseline (on January 1, 2010).
(b) Exclusion: participants who reported having heart disease or stroke during health screenings between 2006 and 2009, those who had an estimated glomerular filtration rate of <30 ml/min/1.73 m^2 in 2009 health screening, and those who died or in whom a critical cardiovascular event occurred before January 1, 2010

© 2019 Jung HH. *JAMA Network Open.*

eMethods 3. Covariates

Outlier data were excluded from the health screening records.
(a) systolic BP <90 mm Hg or >200 mm Hg
(b) diastolic BP <30 mm Hg or >140 mmHg
(c) blood glucose <30 mg/dl or >900 mg/dl
(d) total cholesterol <130 mg/dl or >320 mg/dl
(e) HDL cholesterol <20 mg/dl or >100 mg/dl
(f) serum creatinine <0.3 mg/dl or >15.0 mg/dl
(g) body mass index <10 or >50

Treated and untreated BP records were collected separately.
(a) if antihypertensive prescription ≥90 days in the year of BP measurement, the BP → treated BP
(b) if not, the BP → untreated BP

Income levels were determined by income based insurance contributions.
The amounts of alcohol consumption were calculated as the number of drinks averaged per day.

In each year of follow-up, a new average was calculated for each time period as follows.
(a) primary cohort: 2002–2005, 2002–2006, 2002–2007, 2002–2008, 2002–2009, 2002–2010, 2002–2011, 2002–2012, 2002–2013, and 2002–2014
(b) secondary cohort: 2006–2009, 2006–2010, 2006–2011, 2006–2012, 2006–2013, 2006–2014, 2006–2015, and 2006–2016

Using the yearly updated values, the variables were categorized as follows.
(a) systolic BP: <110, 110–119, 120–129, 130–139, 140–149, 150–159, or ≥160 mm Hg
(b) diastolic BP: <60, 60–69, 70–79, 80–89, 90–99, or ≥100 mm Hg
(c) alcohol consumption: 0.0, 0.1–0.4, 0.5–1.4, 1.5–2.9, or ≥3.0 drinks per day
(d) exercise frequency: <1, 1–2, 3–4, or ≥5 days per week
(e) body mass index: <18.5, 18.5–22.9, 23.0–24.9, 25.0–29.9, or ≥30.0 kg/m^2

In each year of follow-up, the status of health conditions were determined.
(a) diabetes mellitus: yes or no
(b) hyperlipidemia: yes or no
(c) proteinuria: yes or no
(d) smoking: never, former, or active smoker
(e) antihypertensive compliance: regular, irregular, or never use

Antihypertensive compliance was determined as follows.
Initiation of antihypertensive treatment was defined as the first year when antihypertensive drugs were prescribed for ≥90 days per year. Among cases that initiated treatment, regular use was considered when antihypertensives were prescribed for >half of each follow-up period (from initiation of treatment to each year of follow-up), irregular use was when the prescription was ≤half of each follow-up period, and never use when the prescription was never for ≥90 days per year.

Using baseline data, the variables were categorized as follows.
(a) age: 40–44, 45–49, 50–54, 55–59, 60–64, 65–69, 70–74, and 75–79 years
(b) sex: male or female
(c) family history of cardiovascular disease: yes or no

Categories for missing values were included for all variables to minimize the loss of cases in the analysis.

© 2019 Jung HH. *JAMA Network Open.*

eMethods 4. Risk Categories

Both cohort participants were grouped into three risk categories by the number of risk factors present at baseline (≥3, 2, or ≤1 risk factors). The primary cohort participants were additionally categorized by using the SCORE system (≥7.5%, 2.5–7.4%, or <2.5%) and the WHO/ISH score (≥20%, 10–19%, or <10%). The secondary cohort participants were also categorized by using a Korean prediction model (≥15%, 7.5–14%, or <7.5%) and the Framingham score (≥30%, 15–29%, or <15%).

A. Determination of risk factors in both cohorts

Five risk factors (hypertension, diabetes mellitus, hyperlipidemia, proteinuria, and active smoking) were identified, using the results of health screenings and information on the prescription of drugs. Information on the prescription of drugs in the reimbursement records were captured using NHIS billing codes (eTable 2).

B. In the primary cohort, the 10-year risk of cardiovascular disease was calculated with following variables.

(a) Age: age (year) in 2005
(b) SBP: time averaged values of treated and untreated systolic BP (mm Hg) for 2002–2005
(c) Total-C: time averaged values of total cholesterol (mg/dl) for 2002–2005
(d) Cig: smoking status (yes or not) on January 1, 2006
(e) DM: diabetes status (yes or not) on January 1, 2006

B-1. Calculation of SCORE risk in the primary cohort

The SCORE risk was calculated according to 2003 SCORE paper,[4] using formulas for low-risk regions.

```
In males, α_CHD=-22.1, p_CHD=4.71, α_nonCHD=-26.7, p_nonCHD=5.64
In females, α_CHD=-29.8, p_CHD=6.36, α_nonCHD=-31.0, p_nonCHD=6.62

βchol_CHD=0.24, βsbp_CHD=0.018, βcig_CHD=0.71
βchol_nonCHD=0.02, βsbp_nonCHD=0.022, βcig_nonCHD=0.63

CHD_S0_age = exp(-(exp(α_CHD)) * ((Age - 20)**p_CHD))
CHD_S0_age_10 = exp(-(exp(α_CHD)) * ((Age - 10)**p_CHD))
CHD_ω = (βchol_CHD * (Total-C * 0.0259 - 6)) + (βsbp_CHD * (SBP - 120)) + (βcig_CHD * Cig)
CHD_S_age = CHD_S0_age**exp(CHD_ω)
CHD_S_age_10 = CHD_S0_age_10**exp(CHD_ω)
CHD_S10_age = (CHD_S_age_10 / CHD_S_age)
Risk10_CHD = (1 - CHD_S10_age)

nonCHD_S0_age = exp(-(exp(α_nonCHD)) * ((Age-20)**p_nonCHD))
nonCHD_S0_age_10 = exp(-(exp(α_nonCHD)) * ((Age-10)**p_nonCHD))
nonCHD_ω = (βchol_nonCHD * (Total-C * 0.0259 - 6)) + (βsbp_nonCHD * (SBP - 120)) + (βcig_nonCHD *
Cig)
nonCHD_S_age = nonCHD_S0_age**exp(nonCHD_ω)
nonCHD_S_age_10 = nonCHD_S0_age_10**exp(nonCHD_ω)
nonCHD_S10_age = (nonCHD_S_age_10 / nonCHD_S_age)
Risk10_nonCHD = (1 - nonCHD_S10_age)

In participants without diabetes, SCORE risk score = 100 * (Risk10_CHD + Risk10_nonCHD)
In males with diabetes, SCORE risk score = 200 * (Risk10_CHD + Risk10_nonCHD)
In females with diabetes, SCORE risk score = 400 * (Risk10_CHD + Risk10_nonCHD)
```

B-2. Calculation of WHO/ISH risk scores in the primary cohort

The scores were calculated using a single comma delimited file extracted from WHO/ISH cardiovascular risk assessment charts.[5]

C. In the secondary cohort, the 10-year risk of cardiovascular disease was calculated with following variables.

(a) Age: age (year) in 2009
(b) SBP: time averaged values of treated and untreated systolic BP (mm Hg) for 2006–2009
(c) Total-C: time averaged values of total cholesterol (mg/dl) for 2006–2009
(d) HDL-C: HDL cholesterol (mg/dl) in 2009

© 2019 Jung HH. *JAMA Network Open.*

(e) Cig: smoking status (yes or not) on January 1, 2010
(f) DM: diabetes status (yes or not) on January 1, 2010

C-1. Calculation of local risk scores in the secondary cohort
The scores were calculated according to a Korean prediction model,[6] which was developed on the basis of 2013 ACC/AHA risk score.

In males, KRiskFactor =
(ln(Age) * 9.362) + (((ln(Age))**2) * 2.425)
+ (ln(Total-C) * 6.409) - (ln(Age) * ln(Total-C) * 1.430)
- (ln(HDL-C) * 3.843) + (ln(Age) * ln(HDL-C) * 0.810)
+ (Cig * 2.464) - (ln(Age) * Cig * 0.503)
+ (DM * 0.410)
+ (ln(treated SBP) * 18.589) - (ln(Age) * ln(treated SBP) * 4.116)
+ (ln(untreated SBP) * 18.541) - (ln(Age) * ln(untreated SBP) * 4.112)

In males, Korean risk score = 100 * (1 - 0.96427**exp(KRiskFactor - 87.556))

In females, KRiskFactor =
(ln(Age) * - 9.519) + (((ln(Age))**2) * 3.417)
+ (ln(Total-C) * 0.320)
- (ln(HDL-C) * 0.476)
+ (Cig * 0.415)
+ (DM * 0.424)
+ (ln(treated SBP) * 13.402) - (ln(Age) * ln(treated SBP) * 2.889)
+ (ln(untreated SBP) * 13.291) - (ln(Age) * ln(untreated SBP) * 2.876)

In females, Korean risk score = 100 * (1 - 0.96963**exp(KRiskFactor - 24.881))

C-2. Calculation of Framingham risk scores in the secondary cohort
The scores were calculated according to 2008 Framingham paper.[7]

In males,
if antihypertensive prescription ≥90 days, BPfactor = 1.99881 and SBP = treated SBP; if not, BPfactor = 1.93303 and SBP = untreated SBP
if active smoker, Cig = 0.65451; if not, Cig = 0
if diabetes present, DM = 0.57367; if not, DM = 0
RiskFactor = (ln(Age) * 3.06117) + (ln(Total-C) * 1.12370) - (ln(HDL-C) * 0.93263) + (ln(SBP) * BPfactor) + Cig + DM - 23.9802

In males, Framingham risk score = 100 * (1 - 0.88936 ** exp(RiskFactor))

In females,
if antihypertensive prescription ≥90 days in 2009, BPfactor = 2.82263 and SBP = treated SBP; if not, BPfactor = 2.76157 and SBP = untreated SBP
if active smoker, Cig = 0.52873; if not, Cig = 0
if diabetes present, DM = 0.69154; if not, DM = 0
RiskFactor = (ln(Age) * 2.32888) + (ln(Total-C) * 1.20904) - (ln(HDL-C) * 0.70833) + (ln(SBP) * BPfactor) + Cig + DM - 26.1931

In females, Framingham risk score = 100 * (1 - 0.95012 ** exp(RiskFactor))

eMethods 5. Outcomes

Previous studies using the disease codes listed in NHIS reimbursement records have reported that 73%~93% of the disease codes for myocardial infarction or stroke are valid.[6,8] In addition to the disease codes, I used information on revascularization procedures, prescribed peritoneal dialysates, hemodialysis, and kidney transplantation, which were captured by NHIS billing codes, to identify outcomes more reliably.

A. Critical cardiorenal event

Critical cardiorenal event was identified through December 31, 2015 in the primary cohort and through December 31, 2107 in the secondary cohort.
(a) Information on critical care unit admission were captured using NHIS code for admission (NHIS clause code, 02) to critical care unit (NHIS item code, 03). Critical care unit admission from cardiorenal diseases (ICD-10, I00–I99 and N18) was verified with the primary medical diagnosis listed in reimbursement records.
(b) Information on revascularization procedures of coronary, cerebral, and carotid arteries were captured using NHIS billing codes (eTable 3). Revascularization for myocardial infarction (ICD-10, I21– I22) or stroke (ICD-10, I63–I64) was verified with the primary diagnosis listed in reimbursement records.
(c) Information on hemodialysis (NHIS billing code, O7020 and O9991), prescribed peritoneal dialysates (eTable 3), and kidney transplantation (NHIS billing code, R3280; and ICD-10, Z94.0) were captured from NHIS reimbursement records. Using the information, end-stage kidney disease with dialysis for ≥90 days per year or kidney transplantation was verified.

B. All-cause death

All-cause death was confirmed through December 31, 2015 in the primary cohort and through December 31, 2017 in the secondary cohort, using information on date of death, which was included in eligibility database of the NHID.

© 2019 Jung HH. *JAMA Network Open.*

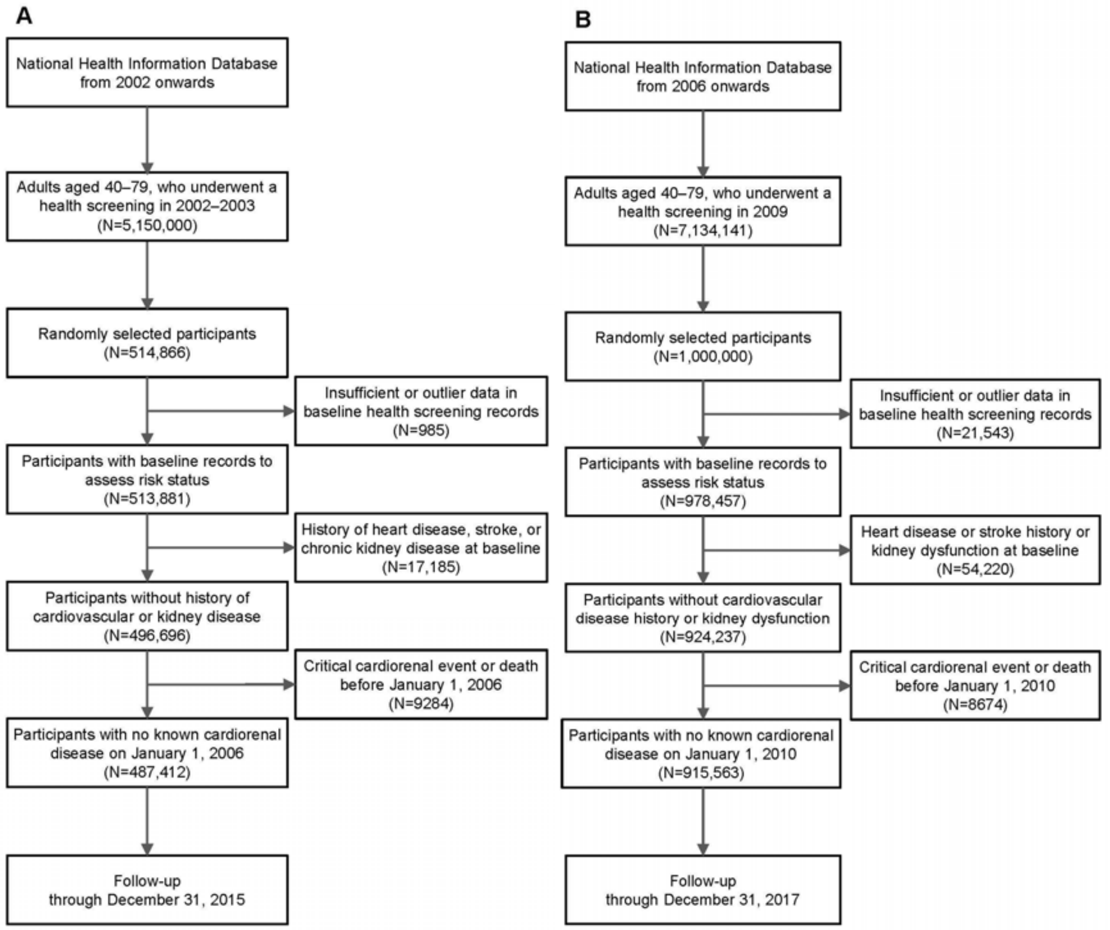

eFigure 1. Flow Charts of Participant Selection in The Primary (A) and Secondary (B) Cohorts

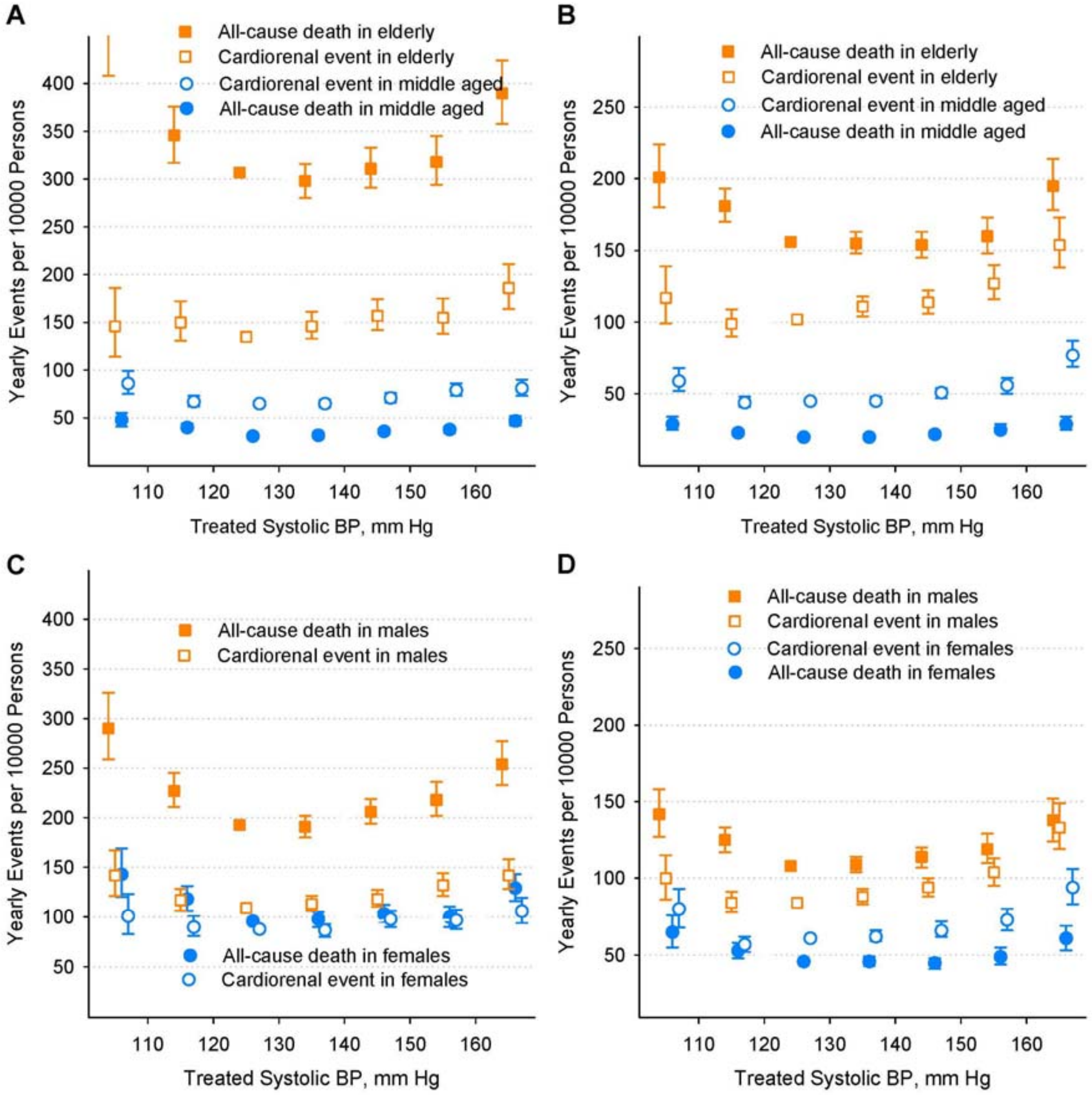

eFigure 2. Yearly Event Rates in Age- or Sex-Stratified Subgroups

The 1-year rates in the subgroups stratified by baseline age (<65 years or ≥65 years) or sex were estimated in the primary (A and C) and secondary (B and D) cohorts, by multiplying the hazard ratios by the mean of the age specific rates in the reference group (systolic BP, 120–129 mm Hg). All analyses were adjusted for age, sex, family history of cardiovascular disease, income level, smoking, alcohol consumption, exercise frequency, body mass index, diabetes, hyperlipidemia, and proteinuria, except for sex in sex-stratified analyses. The critical cardiorenal event was a composite of admission to critical care unit with cardiovascular or chronic kidney disease, revascularization for myocardial infarction or stroke, and new onset end-stage kidney disease. Error bars indicate 95% CIs. BP, blood pressure.

© 2019 Jung HH. *JAMA Network Open.*

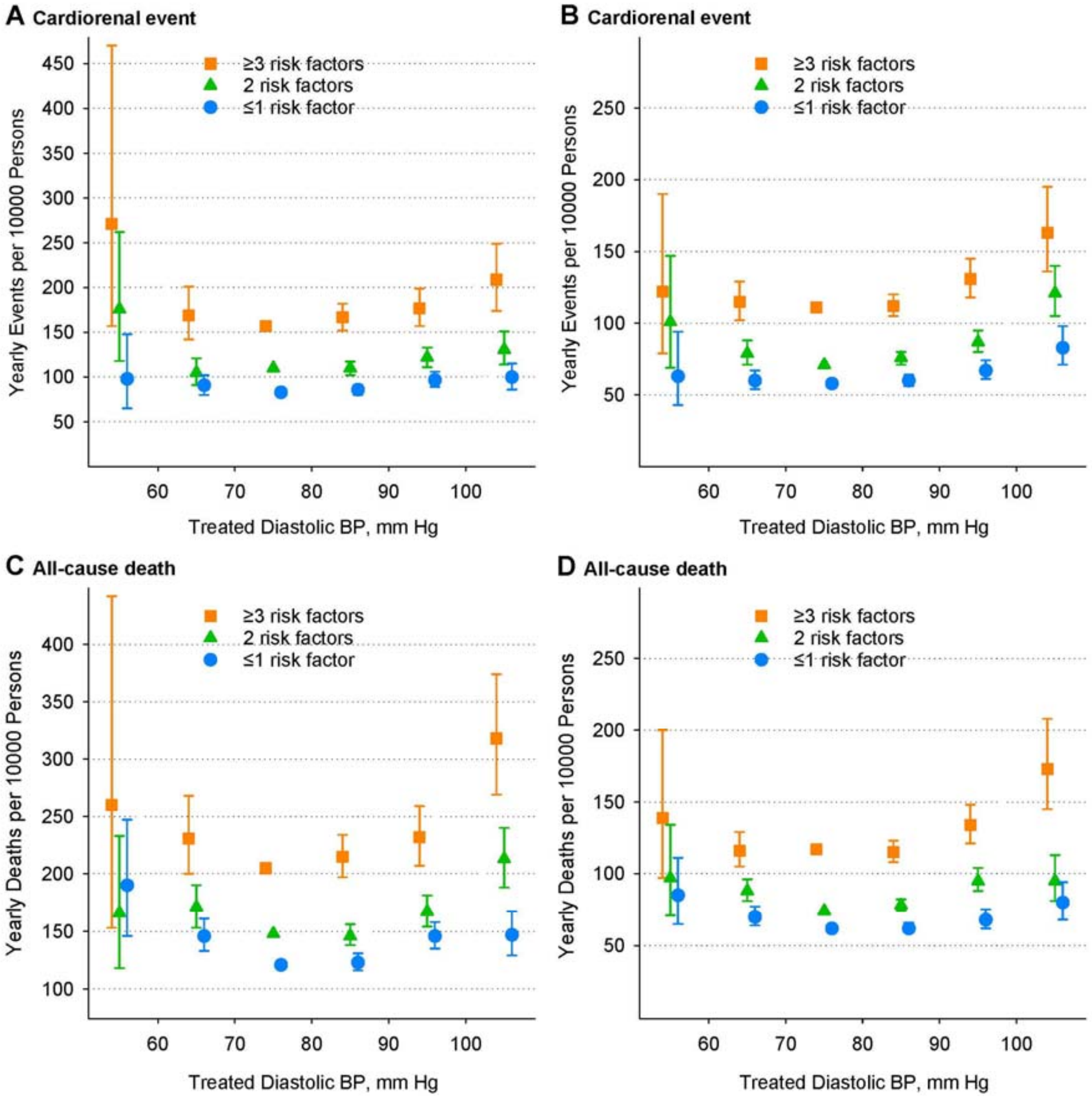

eFigure 3. Yearly Event Rates According to Treated Diastolic BP and Risk Categories
The 1-year rates were estimated in the primary (A and C) and secondary (B and D) cohorts, by multiplying the hazard ratios by the mean of the age specific rates in the reference group (diastolic BP, 70–79 mm Hg). All analyses were adjusted for age, sex, family history of cardiovascular disease, income level, smoking, alcohol consumption, exercise frequency, body mass index, diabetes, hyperlipidemia, and proteinuria. The critical cardiorenal event was a composite of admission to critical care unit with cardiovascular or chronic kidney disease, revascularization for myocardial infarction or stroke, and new onset end-stage kidney disease. Error bars indicate 95% CIs. BP, blood pressure. WHO/ISH, World Health Organization/International Society of Hypertension.

© 2019 Jung HH. *JAMA Network Open.*

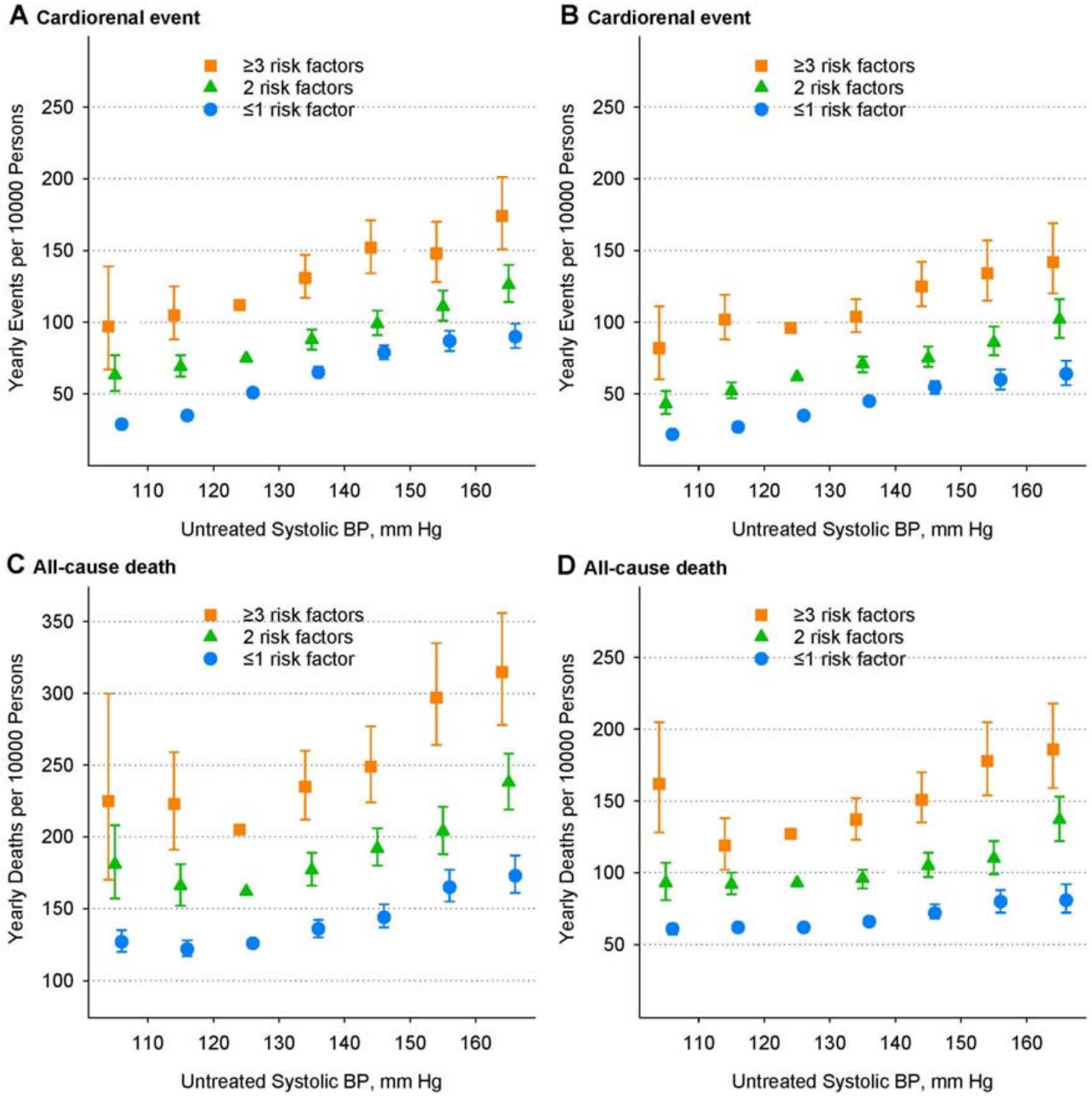

eFigure 4. Yearly Event Rates According to Untreated Systolic BP and Risk Categories

The 1-year rates were estimated in the primary (A and C) and secondary (B and D) cohorts, by multiplying the hazard ratios by the mean of the age specific rates in the reference group (systolic BP, 120–129 mm Hg). All analyses were adjusted for age, sex, family history of cardiovascular disease, income level, smoking, alcohol consumption, exercise frequency, body mass index, diabetes, hyperlipidemia, and proteinuria. The critical cardiorenal event was a composite of admission to critical care unit with cardiovascular or chronic kidney disease, revascularization for myocardial infarction or stroke, and new onset end-stage kidney disease. Error bars indicate 95% CIs. BP, blood pressure.

© 2019 Jung HH. *JAMA Network Open.*

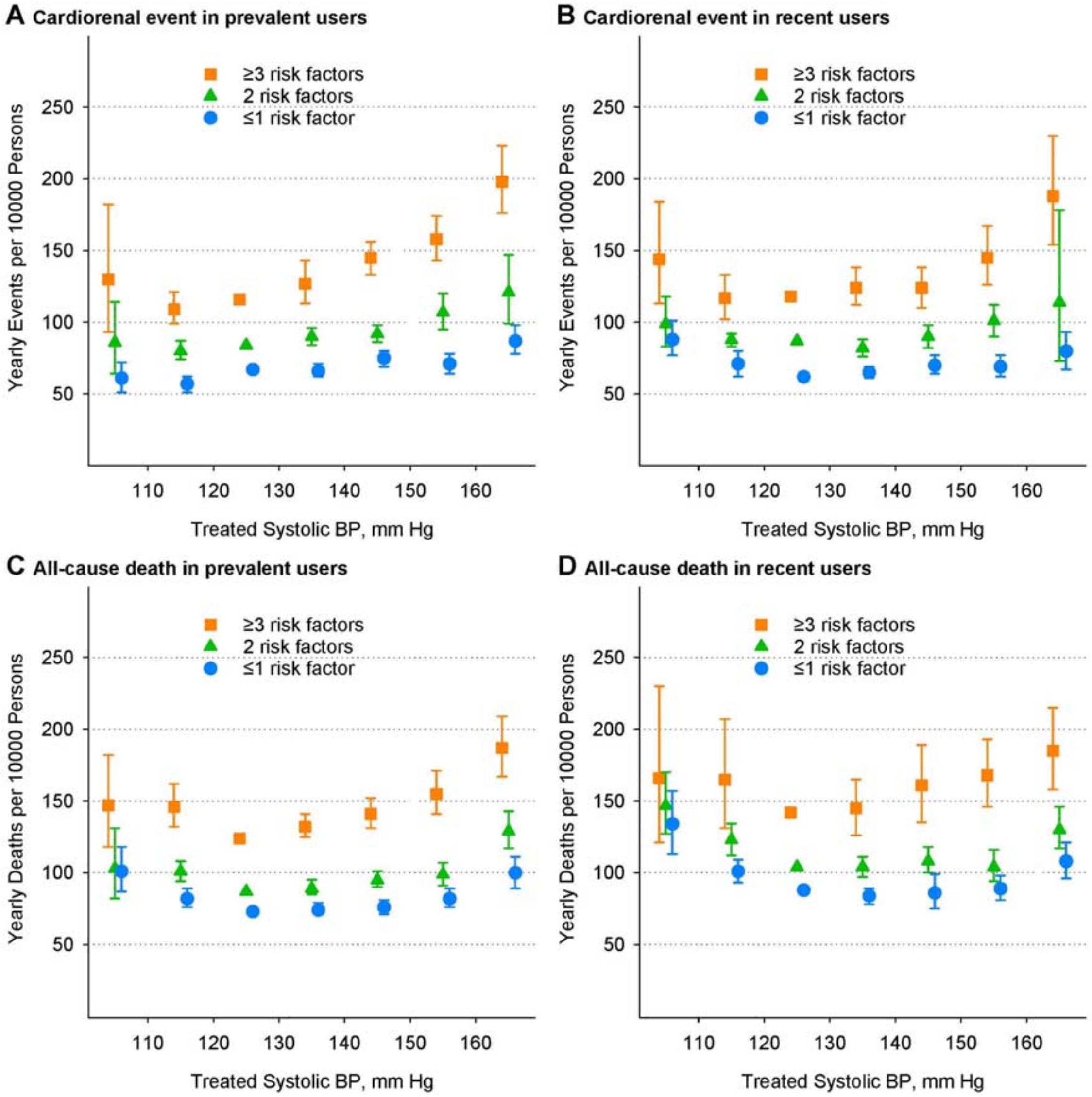

eFigure 5. Yearly Event Rates in Prevalent (A and C) or Recent (B and D) Antihypertensive Users

The 1-year rates were estimated by multiplying the combined hazard ratios by the combined mean of the age specific rates in the reference group (systolic BP, 120–129 mm Hg). The summary effects and 95% CIs of the primary and secondary cohorts were calculated by using the DerSimonian-Laird random-effects model. All analyses were adjusted for age, sex, family history of cardiovascular disease, income level, smoking, alcohol consumption, exercise frequency, body mass index, diabetes, hyperlipidemia, and proteinuria. The critical cardiorenal event was a composite of admission to critical care unit with cardiovascular or chronic kidney disease, revascularization for myocardial infarction or stroke, and new onset end-stage kidney disease. Error bars indicate 95% CIs. BP, blood pressure.

© 2019 Jung HH. *JAMA Network Open.*

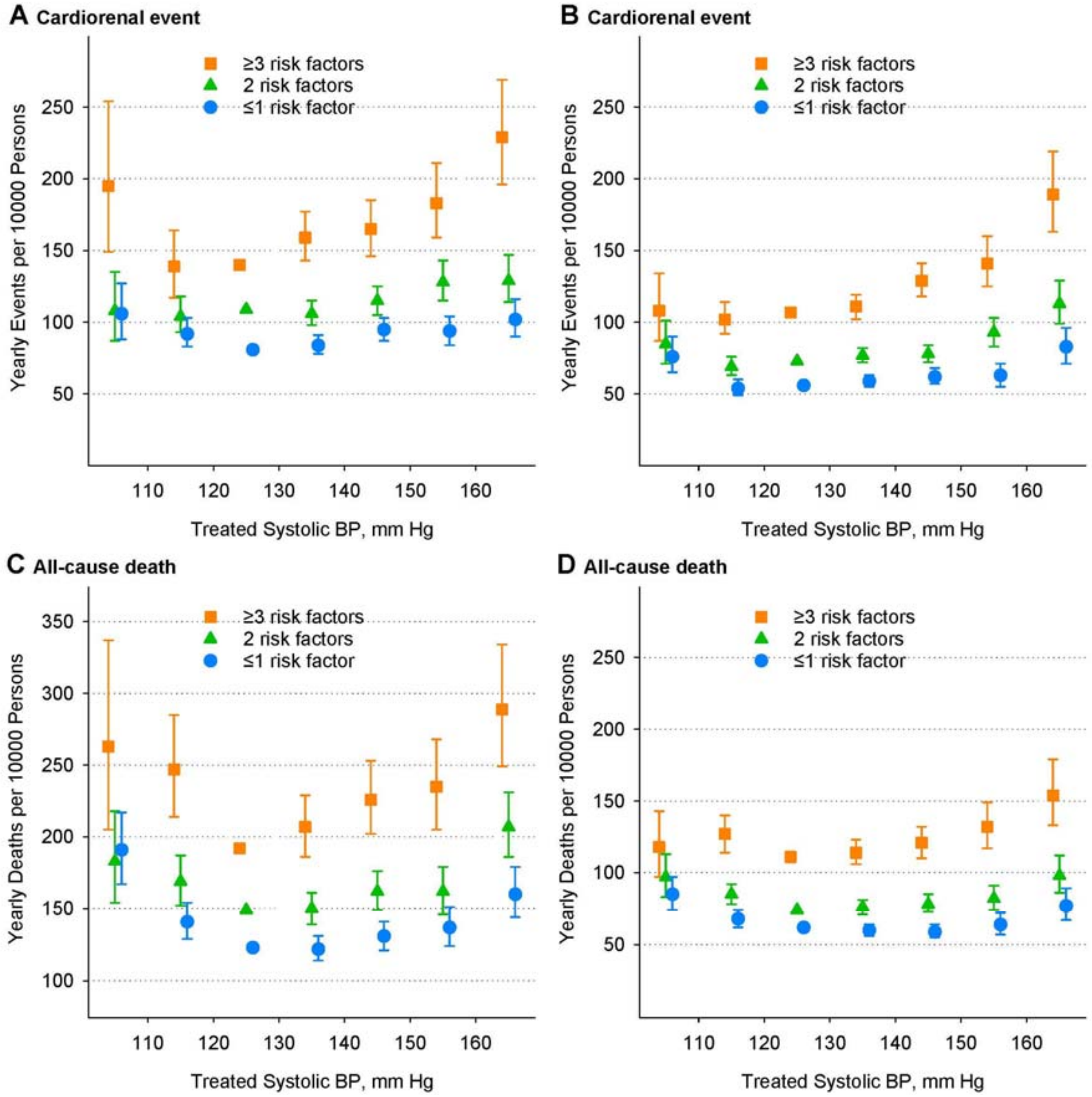

eFigure 6. Yearly Event Rates After Further Adjustment for Compliance

The 1-year rates were estimated in the primary (A and C) and secondary (B and D) cohorts, by multiplying the hazard ratios by the mean of the age specific rates in the reference group (systolic BP, 120–129). The analyses were adjusted for age, sex, family history of cardiovascular disease, income level, smoking, alcohol consumption, exercise frequency, body mass index, diabetes, hyperlipidemia, and proteinuria, and further adjusted for antihypertensive compliance (regular use, irregular use, and nonuse). The critical cardiorenal event was a composite of admission to critical care unit with cardiovascular or chronic kidney disease, revascularization for myocardial infarction or stroke, and new onset end-stage kidney disease. Error bars indicate 95% CIs. BP, blood pressure.

© 2019 Jung HH. *JAMA Network Open.*

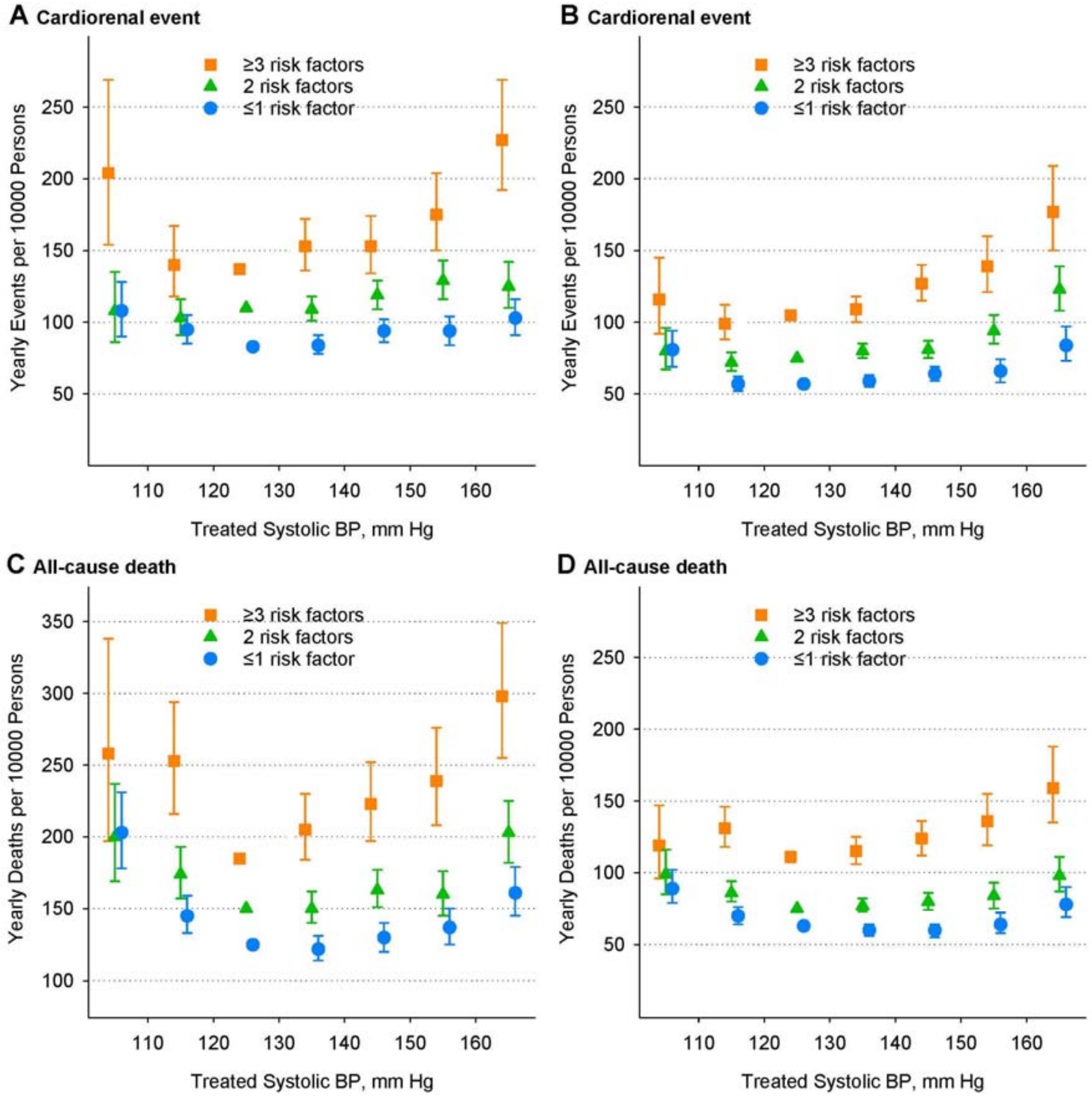

eFigure 7. Yearly Event Rates in Risk Categories Grouped by Risk Factors After Exclusion of Proteinuria

The risk categories were grouped by the number of risk factors present at baseline after exclusion of proteinuria: i.e., ≥3, 2, ≤1 of the four risk factors (hypertension, diabetes, hyperlipidemia, and smoking). Among a total of 487,412 primary cohort participants, 34,050 (7.0%), 110,023 (22.6%), and 343,339 (70.4%) had ≥3, 2, and ≤1 risk factors, respectively. Among a total of 915,563 secondary cohort participants, 65,631 (7.2%), 188,669 (20.6%), and 661,263 (72.2%) had ≥3, 2, and ≤1 risk factors, respectively. The 1-year rates were estimated in the primary (A and C) and secondary (B and D) cohorts, by multiplying the hazard ratios by the mean of the age specific rates in the reference group (systolic BP, 120–129). The analyses were adjusted for age, sex, family history of cardiovascular disease, income level, smoking, alcohol consumption, exercise frequency, body mass index, diabetes, hyperlipidemia, and proteinuria, and further adjusted for antihypertensive compliance (regular use, irregular use, and nonuse). The critical cardiorenal event was a composite of admission to critical care unit with cardiovascular or chronic kidney disease, revascularization for myocardial infarction or stroke, and new onset end-stage kidney disease. Error bars indicate 95% CIs. BP, blood pressure.

© 2019 Jung HH. *JAMA Network Open.*

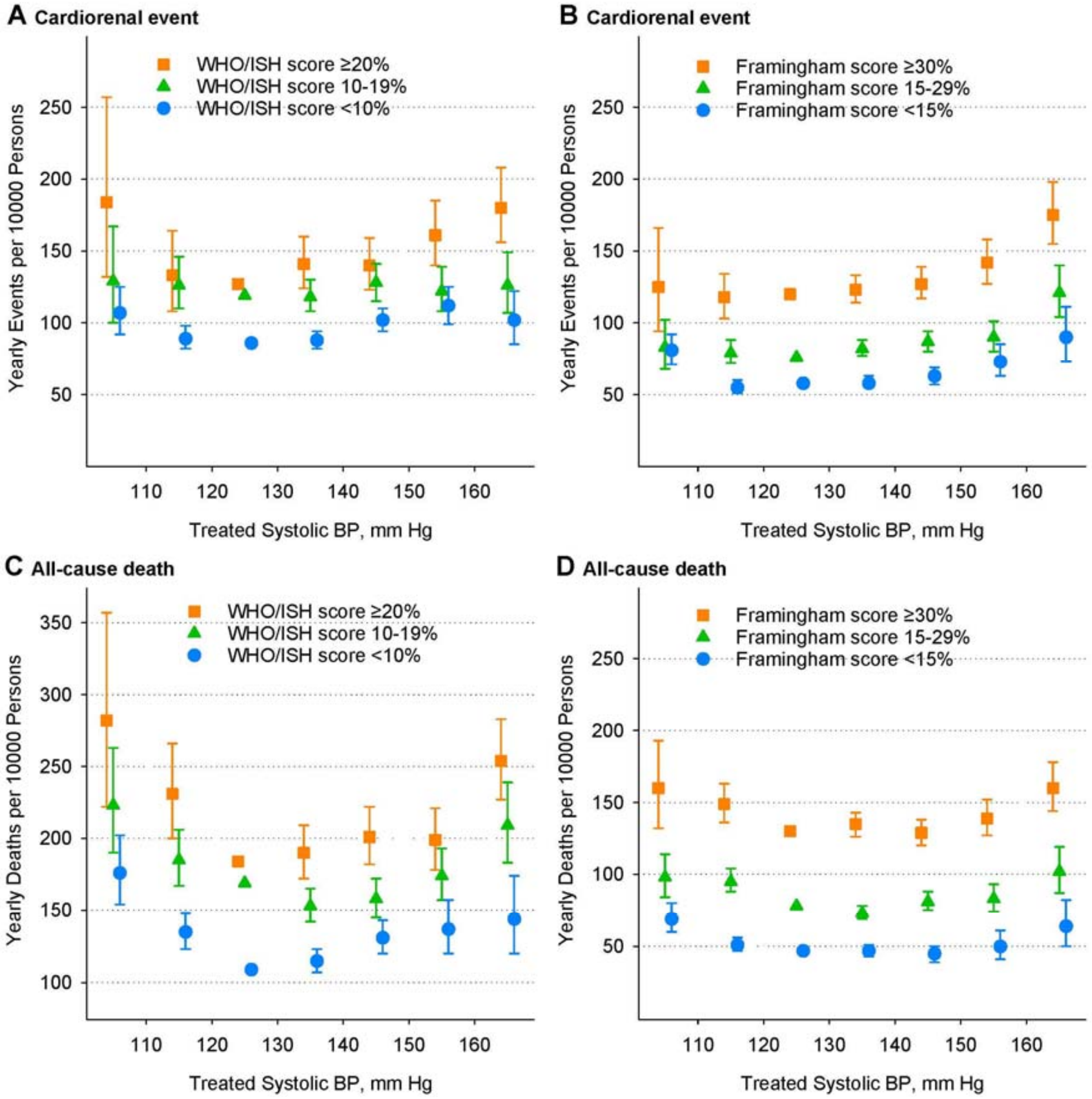

eFigure 8. Yearly Event Rates According to WHO/ISH or Framingham Scores

The 1-year rates were estimated in the primary (A and C) and secondary (B and D) cohorts, by multiplying the hazard ratios by the mean of the age specific rates in the reference group (systolic BP, 120–129 mm Hg). All analyses were adjusted for age, sex, family history of cardiovascular disease, income level, smoking, alcohol consumption, exercise frequency, body mass index, diabetes, hyperlipidemia, and proteinuria. The critical cardiorenal event was a composite of admission to critical care unit with cardiovascular or chronic kidney disease, revascularization for myocardial infarction or stroke, and new onset end-stage kidney disease. Error bars indicate 95% CIs. BP, blood pressure; WHO/ISH, World Health Organization/International Society of Hypertension.

eTable 1. Time-Lagged Covariates for Subsequent Years

Outcome Variable	Time-Varying Covariate		Fixed Covariate
**Clinical Endpoint[a]**	**Yearly Updated Categories[b]**	**Yearly Determined Status[c]**	**Baseline Categories[d]**
	**Primary Cohort**		
2006	2002–2005	2005	2002–2005
2007	2002–2006	2006	2002–2005
2008	2002–2007	2007	2002–2005
2009	2002–2008	2008	2002–2005
2010	2002–2009	2009	2002–2005
2011	2002–2010	2010	2002–2005
2012	2002–2011	2011	2002–2005
2013	2002–2012	2012	2002–2005
2014	2002–2013	2013	2002–2005
2015	2002–2014	2014	2002–2005
	**Secondary Cohort**		
2010	2006–2009	2009	2006–2009
2011	2006–2010	2010	2006–2009
2012	2006–2011	2011	2006–2009
2013	2006–2012	2012	2006–2009
2014	2006–2013	2013	2006–2009
2015	2006–2014	2014	2006–2009
2016	2006–2015	2015	2006–2009
2017	2006–2016	2016	2006–2009

[a] Critical cardiorenal event or all-cause mortality.
[b] Treated and untreated systolic blood pressure, income, body mass index, exercise frequency, and alcohol consumption.
[c] Diabetes, hyperlipidemia, proteinuria, and smoking.
[d] Age, sex, and family history of cardiovascular disease.

© 2019 Jung HH. *JAMA Network Open.*

eTable 2. NHIS Generic Name Codes for Antidiabetics, Antihypertensives, and Statins[a]

**Antidiabetic**	**NHIS Generic Name Code**
Metformin	1915, (4211, 4434, 4435, 4719, 4742, 4743, 4972, 4986), (5188, 6319, 6321, 6372, 6449), (4527, 4529, 4612, 4691, 4718, 4981, 6538, 6539, 6540, 6557), (5023, 5029, 5070, 5071, 5137, 5185, 5186, 5196, 5205, 5206, 5207, 5238, 5247, 6320, 6356, 6357, 6418, 6419, 6420, 6450, 6484, 6485, 6486, 6499, 6500, 6501, 6541), (6398, 6414), (5236, 5237)
Sulfonylurea	1320, 1654, 1655, 1656, 1657, 1658, 1659, 4174, (4211, 4434, 4435, 4719, 4742, 4743, 4972, 4986), (4888, 4889, 4890),
Meglitinide	3795, 4302, 4861, (5188, 6319, 6321, 6372, 6449)
Glitazone	3480, 4319, 5259, (4527, 4529, 4612, 4691, 4718, 4981, 6538, 6539, 6540, 6557), (4888, 4889, 4890), (6303, 6304, 6305, 6306)
DPP-4 inhibitor	5008, 5011, 6133, 6164, 6191, 6242, 6273, 6396, 6453, (5023, 5029, 5070, 5071, 5137, 5185, 5186, 5196, 5205, 5206, 5207, 5238, 5247, 6320, 6356, 6357, 6418, 6419, 6420, 6450, 6484, 6485, 6486, 6499, 6500, 6501, 6541), (6303, 6304, 6305, 6306)
SGLT2 inhibitor	5273, 6282, 6361
GLP-1 agonist	5121, 6266, 6397, 6445, (6398, 6414)
α-glucosidase inhibitor	1006, 2490, 4062, (5236, 5237)
Insulin	1183, 1701, 1702, 1703, 1704, 1705, 1706, 1752, 1753, 2156, 2157, 3278, 4413, 4618, 4849, 4887, 5074, 6268
**Antihypertensive**	**NHIS Generic Name Code**
Thiazide	1708, 1744, 2447, 2627, 2628, 2630, 4513, (2620, 2621, 2626, 4273, 4274, 4554, 4555, 4698, 4699, 4700), (2622, 2623, 3787, 4403, 4407, 4408, 4486, 4487, 4536, 4537, 4901, 4979, 4992, 4993, 5562), (2625, 3564, 3789, 3857, 3858, 4237, 4426, 4432, 4433, 4605, 4774, 4869, 4891, 5026, 5136, 5220, 5268)
β blocker	1079, 1114, 1168, 1170, 1179, 1250, 1370, 1802, 2023, 2098, 4601, 4602, 4831, 4895, (2620, 2621, 2626, 4273, 4274, 4554, 4555, 4698, 4699, 4700), (2624)
α antagonist	1200, 1491, 1749, 2168, 2355, 2629, 4414, 4834
Central sympatholytic	1365, 1927, 1975, 2231, 2631
ACE inhibitor	1042, 1147, 1229, 1330, 1409, 1516, 1635, 1734, 1845, 1968, 2113, 2219, 2224, 2310, 2350, 2351, 2424, 3859, 4028, 5016, 5104, (2622, 2623, 3787, 4403, 4407, 4408, 4486, 4487, 4536, 4537, 4901, 4979, 4992, 4993, 5562), (4470, 4471, 4472, 4660)
AG II antagonist	1226, 1773, 1857, 2471, 3788, 4292, 4685, 5152, 5209, (2625, 3564, 3789, 3857, 3858, 4237, 4426, 4432, 4433, 4605, 4774, 4869, 4891, 5026, 5136, 5220, 5268), (4928, 4929, 4958, 5005, 5006, 5027, 5030, 5115, 5116, 5117, 5139, 5197, 5198, 5199, 5200, 5201, 5212, 5213, 5214, 5222, 5223, 5224, 5226, 5227, 5228, 5229, 5230, 5231, 5232, 5233, 5234, 5475, 5476, 5477, 5478, 5479, 5480, 5822, 5824, 6231, 6294, 6295, 6296, 6313, 6328, 6329, 6330, 6374, 6375, 6376, 6448, 6519, 6520, 6521, 6527, 6529, 6530, 6531), (5240, 5241, 5270, 5271), (5250, 5251, 5252, 5253, 5263, 5264, 5265, 5269, 6297, 6298, 6299, 6300, 6301, 6302, 6316, 6317, 6441, 6442), (6349, 6350, 6351, 6352)
Calcium antagonist	1076, 1140, 1151, 1331, 1457, 1575, 1789, 1803, 1820, 1880, 2010, 2011, 2017, 2024, 2476, 3562, 4412, 4598, 4599, 4646, 4708, 4723, 4724, 4725, 4762, 4797, 4832, 4865, 4959, 5189, 5282, 6145, (2624), (4470, 4471, 4472, 4660), (4928, 4929, 4958, 5005, 5006, 5027, 5030, 5115, 5116, 5117, 5139, 5197, 5198, 5199, 5200, 5201, 5212, 5213, 5214, 5222, 5223, 5224, 5226, 5227, 5228, 5229, 5230, 5231, 5232, 5233, 5234, 5475, 5476, 5477, 5478, 5479, 5480, 5822, 5824, 6231, 6294, 6295, 6296, 6313, 6328, 6329, 6330, 6374, 6375, 6376, 6448, 6519, 6520, 6521, 6527, 6529, 6530, 6531), (4723, 4724, 4725, 5189, 6145)
Direct vasodilator	1707, 1961, 2293, 4236, 4606, 4714, 5124
**Statin**	**NHIS Generic Name Code**
Atorvastatin	1115, 5022, (6338, 6339, 6348), (5240, 5241, 5270, 5271), (4723, 4724, 4725, 5189, 6145)
Rosuvastatin	4540, (6407, 6408, 6409), (5250, 5251, 5252, 5253, 5263, 5264, 5265, 5269, 6297, 6298, 6299, 6300, 6301, 6302, 6316, 6317, 6441, 6442)
Simvastatin	2278, 4710, 4711, (4710, 4711, 5078), (6314, 6315)
Pravastatin	2166, 5193, (6349, 6350, 6351, 6352)
Pitavastatin	4709
Fluvastatin	1624
Lovastatin	1858, 1859
Cerivastatin	1304

[a] The codes of combination drugs are in parentheses.

ACE, angiotensin-converting-enzyme; AG, angiotensin; DPP-4 dipeptidyl peptidase 4; GLP-1, glucagon-like peptide-1; NHIS, National Health Insurance Service; SGLT2, sodium-glucose transport protein 2.

© 2019 Jung HH. *JAMA Network Open.*

eTable 3. NHIS Codes for Arterial Revascularization Procedures and Peritoneal Dialysates

Procedure	NHIS Billing Code
Percutaneous coronary angioplasty	M6551, M6552, M6553, M6554
Percutaneous coronary stent insertion	M6561, M6562, M6563, M6564, M6565, M6566, M6567
Percutaneous coronary thrombolysis or thrombectomy	M6634, M6638
Percutaneous coronary atherectomy	M6571, M6572
Coronary artery bypass graft	O1647, OA641, OA642
Coronary artery endarterectomy	O1830
Percutaneous cerebral angioplasty	M6593, M6599
Percutaneous cerebral stent insertion	M6601
Percutaneous cerebral thrombolysis or thrombectomy	M6630, M6636
Percutaneous carotid angioplasty	M6594
Percutaneous carotid stent insertion	M6602
Percutaneous carotid thrombolysis or thrombectomy	M6635, M6637
Transluminal carotid atherectomy	O0226
Carotid endarterectomy	O0227
**Drug**	**NHIS generic name code**
Peritoneal dialysate	3214, 3216, 3218, 3220, 3221, 3222, 3223, 3224, 3225, 3240, 3243, 3244, 3245, 3246, 3247, 3248, 3249, 3250, 3251, 3252, 3253, 3254, 3255, 3256, 3257, 3258, 3259, 3260, 3261, 3262, 3263, 3496, 3497, 3498, 3499, 3500, 3501, 3502, 3503, 3506, 3507, 3508, 3509, 3510, 3511, 3512, 3513, 3514, 3515, 3516, 3517, 3518, 3519, 3520, 3521, 3522, 3523, 3524, 3525, 3526, 3527, 3528, 3529, 3530, 3531, 3532, 3533, 3534, 3535, 3536, 3537, 3538, 3539, 3540, 3541, 3601, 3602, 3603, 3629, 3630, 3659, 3660, 3661, 3662, 3663, 3666, 3667, 3668, 3669, 3808, 3809, 4008, 4009, 4010, 4095, 4096, 4097, 4098, 4099, 4229, 4230, 4231, 4232, 4234, 4235, 4308, 4309, 4310, 4311, 4312, 4313, 4490, 4491, 4492, 4498, 4499, 4500, 4504, 4505, 4506, 4507, 4509, 4637, 4638, 4639, 4640, 4641, 4642, 4643, 4644, 4646, 4647, 4648, 4845, 4846, 4847, 4935, 4936, 4949, 4950, 4951, 5096, 5097, 5098, 5107, 5108, 5109, 5110, 5111, 5135, 5161, 5162, 5163

NHIS, National Health Insurance Service.

© 2019 Jung HH. *JAMA Network Open.*

eTable 4. Baseline Characteristics of The Study Participants According to Risk Scores From Cardiovascular Risk Calculators[a]

Characteristic	Primary Cohort			Secondary Cohort		
	**SCORE risk score**			**Korean risk score**		
	<2.5%	2.5–7.4%	≥7.5%	<7.5%	7.5–14%	≥15%
No. of participants	336341[b]	101548[b]	44384[b]	692387	151897	71279
BP, mean (SD), mm Hg						
Systolic BP	122.6 (13.9)	133.6 (15.2)	143.3 (17.0)	121.4 (12.6)	131.4 (13.9)	135.4 (14.8)
Diastolic BP	77.6 (9.7)	82.6 (10.1)	85.1 (10.7)	76.3 (8.8)	80.4 (9.1)	80.6 (9.1)
Age, median (IQR), years[c]	47 (43-52)	60 (55-65)	67 (63-72)	49 (44-54)	65 (61-68)	72 (70-76)
Men, %	48.2%	68.4%	65.4%	47.8%	56.2%	60.1%
Family history of CVD, %	11.4%	8.4%	5.6%	9.7%	7.2%	4.9%
Low income, %	19.8%	26.7%	31.3%	39.5%	41.2%	35.2%
Hypertension, %	39.3%	68.8%	84.6%	30.5%	65.7%	77.8%
Diabetes, %	5.4%	21.4%	49.9%	7.6%	24.5%	40.4%
Hyperlipidemia, %	19.7%	27.9%	31.2%	22.8%	35.1%	34.1%
Proteinuria, %	2.7%	3.8%	5.4%	3.6%	5.3%	6.9%
Active smoker, %	17.3%	29.1%	36.9%	20.1%	24.2%	26.4%
Body mass index ≥30.0, %	2.4%	2.9%	3.1%	2.8%	3.6%	3.1%
Physical inactivity, %	43.2%	46.8%	55.2%	47.2%	54.3%	61.6%
Alcohol ≥3.0 drinks/day, %	7.5%	11.7%	11.4%	10.0%	10.8%	9.4%
	**WHO/ISH risk score**			**Framingham risk score**		
	<10%	10–19%	≥20%	<15%	15–29%	≥30%
No. of participants	383373	66808	37231	537337	224594	85228
BP, mean (SD), mm Hg						
Systolic BP	123.0 (13.1)	136.1 (15.9)	149.4 (18.2)	120.9 (12.4)	131.8 (13.4)	137.6 (14.7)
Diastolic BP	77.8 (9.3)	83.2 (10.5)	88.8 (11.7)	75.8 (8.6)	81.3 (8.8)	82.8 (9.3)
Age, median (IQR), years[c]	48 (43–54)	62 (57–67)	64 (59–69)	49 (44–56)	60 (54–68)	66 (60–72)
Men, %	52.9%	57.8%	58.6%	40.0%	75.4%	88.9%
Family history of CVD, %	11.0%	7.7%	6.6%	9.4%	7.9%	6.7%
Low income, %	20.6%	28.2%	30.2%	39.3%	39.2%	41.7%
Hypertension, %	41.4%	74.1%	91.0%	28.9%	66.8%	84.5%
Diabetes, %	7.7%	21.8%	50.7%	5.1%	27.8%	54.4%
Hyperlipidemia, %	17.9%	34.4%	46.2%	21.8%	36.0%	39.0%
Proteinuria, %	2.8%	4.0%	6.1%	3.3%	5.6%	9.1%
Active smoker, %	19.3%	26.4%	37.1%	14.5%	36.2%	51.5%
Body mass index ≥30.0, %	2.2%	3.4%	4.7%	2.7%	4.0%	3.7%
Physical inactivity, %	43.3%	51.2%	54.2%	48.9%	50.4%	52.8%
Alcohol ≥3.0 drinks/day, %	8.3%	9.7%	12.1%	8.2%	15.0%	16.7%

[a] The risk categories were grouped by the risk scores calculated at baseline (on January 1, 2006 and January 1, 2010 in the primary and secondary cohorts, respectively).
[b] The remaining 5139 had missing SCORE risk score.
[c] The values are the ages at the end of 2005 and 2009 in the primary and secondary cohorts, respectively.
WHO/ISH, World Health Organization/International Society of Hypertension; BP, blood pressure; SD, standard deviation; IQR, interquartile range; CVD, cardiovascular disease; SCORE, Systematic COronary Risk Evaluation.

© 2019 Jung HH. *JAMA Network Open.*

eTable 5. Cardiorenal Event and All-Cause Death According to Systolic BP and Risk Categories[a]

Outcome	No. of Events/No. of Person Years in the Primary Cohort							
	**Treated systolic BP, mm Hg**							
	<110	110–119	120–129	130–139	140–149	150–159	≥160	Missing case
Critical cardiorenal event[b]								
≥3 risk factors	61/3722	190/16542	542/45128	861/60413	564/36280	308/17028	232/10034	1272/163205
2 risk factors	84/9638	363/42424	1062/112611	1461/148754	988/86573	514/38610	310/22633	3010/571846
≤1 risk factor	135/21766	506/77706	1128/174156	1560/207430	1025/109854	469/48446	298/27119	6478/2589089
All-cause death								
≥3 risk factors	71/4053	270/17807	579/48058	906/64178	660/38604	359/17974	278/10664	2036/165718
2 risk factors	146/10239	504/45000	1184/118894	1705/156239	1202/90675	590/40439	480/23506	5441/577216
≤1 risk factor	270/23021	717/81639	1375/181886	1826/216216	1191/114468	618/50224	452/28092	13513/2601458
	**Untreated systolic BP, mm Hg**							
	<110	110–119	120–129	130–139	140–149	150–159	≥160	Missing case
Critical cardiorenal event[b]								
≥3 risk factors	31/3758	161/21417	483/64250	759/83256	629/54974	363/29544	359/23550	1245/71603
2 risk factors	111/24439	470/101483	1121/224446	1539/242380	1190/149467	715/72564	662/55625	1984/162685
≤1 risk factor	543/423008	1583/884155	2847/929337	2356/501953	1305/198090	724/89080	557/60145	1684/169798
All-cause death								
≥3 risk factors	52/3890	234/21978	619/66060	981/86182	807/57279	546/30856	480/24800	1440/76011
2 risk factors	225/24779	778/103120	1703/228480	2287/248020	1769/154025	1055/75464	1003/58068	2432/170252
≤1 risk factor	1521/424647	3671/889449	5018/938896	3779/510638	1883/203067	1103/92016	870/62232	2117/176059
**Outcome**	**No. of Events/No. of Person Years in the Secondary Cohort**							
	**Treated systolic BP, mm Hg**							
	<110	110–119	120–129	130–139	140–149	150–159	≥160	Missing case
Critical cardiorenal event[b]								
≥3 risk factors	91/9032	432/45637	1190/117609	1412/130095	803/60536	327/22123	221/11099	1214/181711
2 risk factors	135/17762	596/90695	1661/232582	2039/257380	967/114821	419/41021	247/19744	3032/681437
≤1 risk factor	169/29809	557/118246	1407/260662	1727/277035	855/121146	316/43667	209/21858	7845/4228662
All-cause death								
≥3 risk factors	112/9411	535/47308	1254/121978	1536/134791	841/63041	341/22985	209/11623	1472/183661
2 risk factors	179/18472	796/93588	1789/239530	2207/264806	1117/118376	434/42305	257/20340	4164/686311
≤1 risk factor	251/31122	782/122033	1648/267746	1922/284504	910/124171	370/44705	229/22406	12772/4242346
	**Untreated systolic BP, mm Hg**							
	<110	110–119	120–129	130–139	140–149	150–159	≥160	Missing case
Critical cardiorenal event[b]								
≥3 risk factors	44/6586	246/32086	528/79693	720/100173	471/52328	240/23257	187/16116	3254/267603
2 risk factors	135/40652	597/152162	1265/281177	1445/271192	763/128575	394/53924	302/33394	4195/494366
≤1 risk factor	749/757329	2252/1508387	3326/1457540	2383/686687	827/168053	354/60146	237/34245	2957/428698
All-cause death								
≥3 risk factors	79/6713	247/32816	605/81338	833/102286	522/53780	284/23953	214/16750	3516/277162
2 risk factors	238/41025	875/153972	1615/284845	1768/275588	998/131014	475/55188	384/34372	4590/507724
≤1 risk factor	1521/759372	4062/1514786	5023/1466582	3228/693565	1030/170424	432/61259	284/35065	3304/437980

[a] The risk categories were grouped by the number of risk factors present at baseline (on January 1, 2006 and January 1, 2010 in the primary and secondary cohorts, respectively): i.e., ≥3, 2, ≤1 of the five risk factors (hypertension, diabetes, hyperlipidemia, proteinuria, and smoking).

[b] The critical cardiorenal event was a composite of admission to critical care unit with cardiovascular or chronic kidney disease, revascularization for myocardial infarction or stroke, and new onset end-stage kidney disease.

BP, blood pressure.

© 2019 Jung HH. *JAMA Network Open.*

eTable 6. Yearly Event Rates According to Achieved BP and Risk Categories in The Primary Cohort[a]

Outcome	Yearly Events (95% Confidence Interval) per 10000 Persons							
	**Treated systolic BP, mm Hg**							
	<110	110–119	120–129[b]	130–139	140–149	150–159	≥160	Missing case
Critical cardiorenal event[c]								
≥3 risk factors	194 (149–254)	139 (117–164)	140	158 (142–176)	163 (145–184)	181 (157–208)	225 (192–263)	115 (103–128)
2 risk factors	110 (88–137)	105 (93–118)	109	106 (97–114)	114 (104–124)	126 (114–141)	126 (111–143)	80 (74–86)
≤1 risk factor	105 (88–126)	92 (83–102)	81	83 (77–90)	93 (86–101)	92 (82–102)	99 (87–112)	58 (54–62)
All-cause mortality								
≥3 risk factors	275 (215–353)	252 (217–291)	192	206 (186–229)	226 (202–253)	235 (206–269)	289 (250–335)	270 (244–298)
2 risk factors	196 (165–233)	172 (155–191)	149	149 (138–160)	161 (148–174)	161 (146–178)	206 (185–230)	192 (179–205)
≤1 risk factor	200 (175–228)	144 (132–158)	123	121 (113–130)	128 (119–139)	134 (122–148)	157 (141–175)	145 (137–154)
	**Untreated systolic BP, mm Hg**							
	<110	110–119	120–129[b]	130–139	140–149	150–159	≥160	Missing case
Critical cardiorenal event[c]								
≥3 risk factors	97 (67–139)	105 (88–125)	112	131 (117–147)	152 (134–171)	148 (128–170)	174 (151–201)	171 (152–194)
2 risk factors	63 (52–77)	69 (62–77)	75	88 (81–95)	99 (91–108)	111 (101–122)	126 (114–140)	112 (103–122)
≤1 risk factor	29 (27–32)	35 (33–38)	51	65 (62–69)	79 (74–84)	87 (80–94)	90 (82–99)	85 (79–92)
All-cause mortality								
≥3 risk factors	225 (170–300)	223 (191–259)	205	235 (212–260)	249 (224–277)	297 (264–335)	315 (278–356)	313 (280–349)
2 risk factors	181 (157–208)	166 (152–181)	162	177 (166–189)	192 (180–206)	204 (188–221)	238 (219–258)	228 (212–245)
≤1 risk factor	127 (120–135)	122 (117–128)	126	136 (130–142)	144 (137–153)	165 (155–177)	173 (161–187)	177 (167–189)
	**Treated diastolic BP, mm Hg**							
	<60	60–70	70–79[b]	80–89	90–99	≥100		Missing case
Critical cardiorenal event[c]								
≥3 risk factors	271 (157–470)	169 (142–201)	157	167 (152–182)	177 (157–199)	209 (174–249)		119 (107–132)
2 risk factors	176 (118–262)	105 (91–121)	110	110 (102–117)	122 (111–133)	131 (114–151)		79 (73–85)
≤1 risk factor	98 (65–148)	91 (80–102)	83	86 (80–91)	97 (89–106)	100 (86–115)		55 (51–58)
All-cause mortality								
≥3 risk factors	260 (153–442)	231 (200–268)	205	215 (197–234)	232 (207–259)	318 (269–374)		265 (243–290)
2 risk factors	166 (118–233)	171 (153–190)	148	146 (138–156)	167 (154–181)	213 (188–240)		184 (174–196)
≤1 risk factor	190 (146–247)	146 (133–161)	121	123 (116–131)	146 (135–158)	147 (129–167)		141 (133–148)
	**Untreated diastolic BP, mm Hg**							
	<60	60–70	70–79[b]	80–89	90–99	≥100		Missing case
Critical cardiorenal event[c]								
≥3 risk factors	145 (65–324)	113 (88–145)	117	126 (113–140)	152 (136–171)	161 (140–186)		168 (150–189)
2 risk factors	53 (29–100)	79 (69–91)	79	88 (82–94)	108 (100–117)	119 (107–131)		111 (102–120)
≤1 risk factor	29 (22–40)	37 (34–39)	47	65 (62–68)	83 (77–88)	89 (81–98)		82 (77–88)
All-cause mortality								
≥3 risk factors	397 (249–635)	238 (197–287)	231	226 (208–247)	290 (263–319)	315 (278–357)		312 (283–345)
2 risk factors	235 (174–318)	170 (153–188)	172	177 (168–187)	206 (193–219)	235 (216–256)		226 (211–242)
≤1 risk factor	130 (111–152)	129 (123–135)	123	137 (132–142)	156 (148–165)	168 (155–183)		172 (162–182)

[a] The 1-year rates were estimated by multiplying the hazard ratios by the mean of the age specific rates in the reference group. All analyses were adjusted for age, sex, family history of cardiovascular disease, income level, smoking, alcohol consumption, exercise frequency, body mass index, diabetes, hyperlipidemia, and proteinuria.
[b] The time averaged systolic BP of 120–129 mm Hg (or the time averaged diastolic BP of 70–80 mm Hg) served as the reference.
[c] The critical cardiorenal event was a composite of admission to critical care unit with cardiovascular or chronic kidney disease, revascularization for myocardial infarction or stroke, and new onset end-stage kidney disease.
BP, blood pressure.

© 2019 Jung HH. *JAMA Network Open.*

eTable 7. Yearly Event Rates According to Achieved BP and Risk Categories in The Secondary Cohort[a]

Outcome	Yearly Events (95% Confidence Interval) per 10000 Persons							
	**Treated systolic BP, mm Hg**							
	<110	110–119	120–129[b]	130–139	140–149	150–159	≥160	Missing case
Critical cardiorenal event[c]								
≥3 risk factors	109 (88–135)	103 (92–115)	107	110 (102–119)	129 (118–141)	141 (125–160)	190 (164–219)	95 (86–104)
2 risk factors	86 (72–103)	70 (64–77)	73	77 (72–82)	78 (72–84)	93 (83–103)	113 (99–129)	62 (57–66)
≤1 risk factor	78 (66–91)	55 (50–60)	56	59 (55–63)	62 (57–67)	63 (55–71)	83 (71–96)	40 (38–43)
All-cause mortality								
≥3 risk factors	121 (99–147)	128 (115–141)	111	114 (106–123)	121 (110–132)	133 (118–150)	157 (135–182)	130 (119–142)
2 risk factors	100 (86–117)	86 (79–93)	74	76 (71–81)	78 (73–84)	82 (74–91)	98 (86–112)	87 (81–93)
≤1 risk factor	87 (76–99)	69 (63–75)	62	59 (56–63)	59 (54–64)	63 (56–71)	75 (65–86)	63 (59–67)
	**Untreated systolic BP, mm Hg**							
	<110	110–119	120–129[b]	130–139	140–149	150–159	≥160	Missing case
Critical cardiorenal event[c]								
≥3 risk factors	82 (60–111)	102 (88–119)	96	104 (93–116)	125 (111–142)	134 (115–157)	142 (120–169)	132 (119–147)
2 risk factors	43 (36–52)	52 (47–58)	62	71 (65–76)	75 (69–83)	86 (77–97)	102 (89–116)	84 (77–91)
≤1 risk factor	22 (20–23)	27 (26–29)	35	45 (42–47)	55 (50–59)	60 (53–67)	64 (56–73)	52 (48–56)
All-cause mortality								
≥3 risk factors	162 (128–205)	119 (102–138)	127	137 (123–152)	151 (135–170)	178 (154–205)	186 (159–218)	161 (146–179)
2 risk factors	93 (81–107)	92 (85–100)	93	96 (89–102)	105 (97–114)	110 (99–122)	137 (122–153)	110 (103–119)
≤1 risk factor	61 (57–64)	62 (59–65)	62	66 (63–69)	72 (68–78)	80 (72–88)	81 (72–92)	72 (68–76)
	**Treated diastolic BP, mm Hg**							
	<60	60–70	70–79[b]	80–89	90–99	≥100		Missing case
Critical cardiorenal event[c]								
≥3 risk factors	122 (79–190)	115 (102–129)	111	112 (105–120)	131 (118–145)	163 (136–195)		94 (86–103)
2 risk factors	101 (69–147)	79 (71–88)	71	76 (71–80)	87 (80–95)	121 (105–140)		60 (56–64)
≤1 risk factor	63 (43–94)	60 (54–67)	58	60 (56–64)	67 (61–74)	83 (71–98)		40 (37–42)
All-cause mortality								
≥3 risk factors	139 (97–200)	116 (105–129)	117	115 (108–123)	134 (121–148)	173 (145–208)		128 (118–140)
2 risk factors	97 (71–134)	88 (81–96)	74	78 (74–82)	95 (88–104)	95 (81–113)		88 (83–94)
≤1 risk factor	85 (65–111)	70 (64–77)	62	62 (59–66)	68 (62–75)	80 (68–94)		63 (60–67)
	**Untreated diastolic BP, mm Hg**							
	<60	60–70	70–79[b]	80–89	90–99	≥100		Missing case
Critical cardiorenal event[c]								
≥3 risk factors	60 (22–161)	110 (91–133)	95	103 (93–114)	129 (115–146)	131 (111–156)		128 (116–142)
2 risk factors	52 (31–87)	52 (46–59)	61	68 (63–73)	78 (71–85)	95 (84–108)		81 (75–88)
≤1 risk factor	28 (22–35)	26 (25–28)	32	43 (41–45)	54 (50–59)	69 (61–79)		49 (46–53)
All-cause mortality								
≥3 risk factors	145 (80–264)	123 (103–147)	133	139 (127–153)	156 (138–175)	188 (160–221)		158 (144–173)
2 risk factors	106 (74–154)	98 (89–108)	93	101 (95–107)	117 (107–126)	139 (123–156)		113 (106–121)
≤1 risk factor	70 (60–82)	59 (57–62)	63	71 (68–73)	74 (69–80)	82 (71–93)		72 (68–76)

[a] The 1-year rates were estimated by multiplying the hazard ratios by the mean of the age specific rates in the reference group. All analyses were adjusted for age, sex, family history of cardiovascular disease, income level, smoking, alcohol consumption, exercise frequency, body mass index, diabetes, hyperlipidemia, and proteinuria.
[b] The time averaged systolic BP of 120–129 mm Hg (or the time averaged diastolic BP of 70–80 mm Hg) served as the reference.
[c] The critical cardiorenal event was a composite of admission to critical care unit with cardiovascular or chronic kidney disease, revascularization for myocardial infarction or stroke, and new onset end-stage kidney disease.
BP, blood pressure.

© 2019 Jung HH. *JAMA Network Open.*

eTable 8. Yearly Event Rates According to Risk Scores From Cardiovascular Risk Calculators[a]

Outcome	Yearly Events (95% Confidence Interval) per 10000 Persons in the Primary Cohort							
	Treated systolic BP, mm Hg							
	<110	110–119	120–129[b]	130–139	140–149	150–159	≥160	Missing case
Critical cardiorenal event[c]								
SCORE risk score ≥7.5%	180 (133–246)	158 (133–187)	150	163 (147–181)	165 (147–184)	181 (160–204)	193 (170–220)	133 (120–148)
SCORE risk score 2.5–7.4%	130 (106–161)	112 (99–126)	103	107 (98–116)	121 (110–132)	121 (108–135)	140 (121–161)	83 (77–90)
SCORE risk score <2.5%	94 (79–113)	79 (71–88)	78	77 (71–83)	85 (78–94)	95 (84–109)	90 (75–109)	47 (44–51)
All-cause mortality								
SCORE risk score ≥7.5%	370 (309–442)	288 (259–320)	257	258 (240–277)	262 (243–283)	264 (243–288)	323 (295–353)	323 (302–345)
SCORE risk score 2.5–7.4%	196 (170–228)	164 (149–180)	136	127 (118–136)	140 (129–152)	151 (135–168)	188 (164–215)	164 (154–175)
SCORE risk score <2.5%	94 (78–115)	73 (64–84)	54	59 (53–66)	65 (57–74)	68 (56–83)	76 (59–99)	68 (61–74)
Critical cardiorenal event[c]								
WHO/ISH score ≥20%	184 (132–257)	133 (108–164)	127	141 (124–160)	140 (123–159)	161 (140–185)	180 (156–208)	123 (108–139)
WHO/ISH score 10–19%	129 (100–167)	126 (110–146)	119	118 (108–130)	128 (115–141)	122 (108–139)	126 (107–149)	88 (80–96)
WHO/ISH score <10%	107 (92–125)	89 (82–98)	86	88 (82–94)	102 (94–110)	112 (99–125)	102 (85–122)	57 (54–61)
All-cause mortality								
WHO/ISH score ≥20%	282 (222–357)	231 (200–266)	184	190 (172–209)	201 (182–222)	199 (178–221)	254 (227–283)	257 (235–282)
WHO/ISH score 10–19%	223 (190–263)	185 (167–206)	169	153 (142–165)	158 (145–172)	174 (157–193)	209 (183–239)	195 (182–209)
WHO/ISH score <10%	176 (154–202)	135 (123–148)	109	115 (107–123)	131 (120–143)	137 (120–157)	144 (120–174)	133 (125–142)

Outcome	Yearly Events (95% Confidence Interval) per 10000 Persons in the Secondary Cohort							
	Treated systolic BP, mm Hg							
	<110	110–119	120–129[b]	130–139	140–149	150–159	≥160	Missing case
Critical cardiorenal event[c]								
Korean risk score ≥15%	166 (129–213)	148 (131–168)	144	155 (143–168)	159 (145–174)	179 (160–200)	224 (197–254)	137 (124–151)
Korean risk score 7.5–14%	99 (81–121)	90 (81–100)	95	94 (88–101)	104 (96–113)	109 (97–122)	137 (119–158)	80 (74–87)
Korean risk score <7.5%	75 (65–86)	52 (48–57)	54	57 (53–61)	60 (55–66)	68 (60–78)	96 (80–114)	35 (33–38)
All-cause mortality								
Korean risk score ≥15%	237 (205–274)	214 (198–232)	185	183 (173–193)	176 (165–188)	185 (170–202)	228 (206–252)	197 (185–209)
Korean risk score 7.5–14%	102 (87–119)	94 (86–102)	77	73 (68–78)	78 (72–85)	86 (76–96)	91 (77–107)	86 (80–92)
Korean risk score <7.5%	54 (46–65)	39 (35–44)	36	37 (34–40)	40 (36–46)	44 (36–53)	59 (46–75)	39 (36–43)
Critical cardiorenal event[c]								
Framingham score ≥30%	125 (94–166)	118 (103–134)	120	123 (114–133)	127 (117–139)	142 (127–158)	175 (155–198)	117 (106–129)
Framingham score 15–29%	83 (68–102)	79 (72–88)	76	82 (77–88)	87 (80–94)	90 (80–101)	121 (104–140)	67 (62–72)
Framingham score <15%	81 (71–92)	55 (51–60)	58	58 (55–63)	63 (57–69)	73 (63–85)	90 (73–111)	38 (36–41)
All-cause mortality								
Framingham score ≥30%	160 (132–193)	149 (136–163)	130	135 (126–143)	129 (120–138)	139 (127–152)	160 (144–178)	149 (139–160)
Framingham score 15–29%	98 (84–114)	95 (88–104)	78	73 (69–78)	81 (75–88)	83 (74–93)	102 (87–119)	86 (80–91)
Framingham score <15%	69 (60–80)	51 (47–56)	47	47 (43–51)	45 (39–50)	50 (41–61)	64 (50–82)	48 (45–52)

[a] The risk categories were grouped by the SCORE and WHO/ISH risk scores and the Korean and Framingham risk scores, in the primary and secondary cohorts, respectively. The 1-year rates were estimated by multiplying the hazard ratios by the mean of the age specific rates in the reference group. All analyses were adjusted for age, sex, family history of cardiovascular disease, income level, smoking, alcohol consumption, exercise frequency, body mass index, diabetes, hyperlipidemia, and proteinuria.

[b] The time averaged systolic BP of 120–129 mm Hg served as the reference.

[c] The critical cardiorenal event was a composite of admission to critical care unit with cardiovascular or chronic kidney disease, revascularization for myocardial infarction or stroke, and new onset end-stage kidney disease.

BP, blood pressure; SCORE, Systematic COronary Risk Evaluation; WHO/ISH, World Health Organization/International Society of Hypertension;

© 2019 Jung HH. *JAMA Network Open.*

eTable 9. Yearly Event Rates in Age- or Sex-Stratified Subgroups[a]

Outcome	Yearly Events (95% Confidence Interval) per 10000 Persons, in the Primary Cohort							
	Treated systolic BP, mm Hg							
	<110	110–119	120–129[b]	130–139	140–149	150–159	≥160	Missing case
Critical cardiorenal event[c]								
Elderly	146 (114–186)	150 (131–172)	135	146 (133–161)	157 (142–174)	155 (138–175)	186 (164–211)	114 (104–125)
Middle-aged	86 (75–99)	67 (62–73)	65	65 (61–69)	71 (66–76)	79 (73–86)	81 (73–90)	45 (43–47)
All-cause mortality								
Elderly	463 (408–525)	346 (317–376)	307	298 (280–316)	311 (291–333)	318 (294–345)	390 (358–424)	373 (352–394)
Middle-aged	48 (41–55)	40 (37–44)	31	32 (30–35)	36 (33–39)	38 (34–41)	47 (42–52)	40 (38–43)
Critical cardiorenal event[c]								
Male	142 (121–167)	117 (106–128)	109	113 (106–121)	118 (110–127)	132 (121–144)	142 (128–158)	83 (78–88)
Female	101 (83–123)	90 (81–101)	88	87 (80–93)	98 (90–106)	97 (88–107)	106 (94–119)	58 (54–62)
All-cause mortality								
Male	290 (259–326)	227 (211–245)	193	191 (180–202)	206 (194–219)	218 (202–236)	254 (233–277)	236 (224–248)
Female	143 (120–169)	118 (106–131)	96	98 (90–105)	103 (95–112)	100 (90–110)	129 (116–143)	117 (109–126)
**Outcome**	**Yearly Events (95% Confidence Interval) per 10000 Persons, in the Secondary Cohort**							
	**Treated systolic BP, mm Hg**							
	<110	110–119	120–129[b]	130–139	140–149	150–159	≥160	Missing case
Critical cardiorenal event[c]								
Elderly	117 (99–139)	99 (90–109)	102	111 (104–118)	114 (106–122)	127 (116–140)	154 (138–173)	87 (80–93)
Middle-aged	59 (52–68)	44 (41–48)	45	45 (43–48)	51 (47–54)	56 (50–61)	77 (69–87)	33 (31–35)
All-cause mortality								
Elderly	201 (180–224)	181 (170–193)	156	155 (148–163)	154 (145–163)	160 (148–173)	195 (178–214)	162 (154–171)
Middle-aged	29 (25–34)	23 (21–25)	20	20 (19–21)	22 (20–24)	25 (23–29)	29 (25–34)	23 (22–25)
Critical cardiorenal event[c]								
Male	100 (86–115)	84 (78–91)	84	88 (83–93)	94 (88–100)	104 (95–113)	133 (119–149)	67 (63–70)
Female	80 (68–93)	57 (52–62)	61	62 (59–66)	66 (62–72)	73 (66–80)	94 (83–106)	43 (40–46)
All-cause mortality								
Male	142 (127–158)	125 (117–133)	108	109 (104–114)	114 (107–120)	119 (110–129)	138 (124–152)	117 (112–123)
Female	65 (55–76)	53 (48–58)	46	46 (43–49)	45 (41–48)	49 (44–55)	61 (53–69)	48 (45–52)

[a] The 1-year rates in the subgroups stratified by baseline age (<65 years or ≥65 years) or sex were estimated by multiplying the hazard ratios by the mean of the age specific rates in the reference group. All analyses were adjusted for age, family history of cardiovascular disease, income level, smoking, alcohol consumption, exercise frequency, body mass index, diabetes, hyperlipidemia, and proteinuria, and the age stratified analyses were further adjusted for sex.

[b] The time averaged systolic BP of 120–129 mm Hg served as the reference.

[c] The critical cardiorenal event was a composite of admission to critical care unit with cardiovascular or chronic kidney disease, revascularization for myocardial infarction or stroke, and new onset end-stage kidney disease.

BP, blood pressure.

© 2019 Jung HH. *JAMA Network Open.*

eTable 10. Yearly Event Rates in Prevalent or Recent Antihypertensive Users[a]

Outcome	Yearly Events (95% Confidence Interval) per 10000 Persons, in the Primary and Secondary Cohorts[b]							
	Treated systolic BP, mm Hg							
	<110	110–119	120–129[c]	130–139	140–149	150–159	≥160	Missing case
Critical cardiorenal event[d] in prevalent users								
≥3 risk factors	130 (93–182)	109 (99–121)	116	127 (113–143)	145 (133–156)	158 (143–174)	198 (176–223)	131 (114–151)
2 risk factors	83 (62–110)	77 (71–84)	84	87 (81–93)	89 (83–95)	103 (91–116)	116 (95–142)	94 (85–105)
≤1 risk factor	65 (55–78)	61 (55–67)	67	72 (67–76)	81 (75–86)	77 (70–84)	94 (84–106)	67 (59–76)
All-cause mortality in prevalent users								
≥3 risk factors	147 (118–182)	146 (132–162)	124	132 (125–141)	141 (131–152)	155 (141–171)	187 (167–209)	184 (127–265)
2 risk factors	103 (82–131)	101 (94–108)	87	89 (85–95)	95 (90–101)	99 (91–107)	129 (117–143)	144 (118–176)
≤1 risk factor	101 (87–118)	82 (76–89)	73	74 (71–79)	76 (71–81)	82 (76–89)	100 (89–111)	107 (86–133)
Critical cardiorenal event[d] in recent users								
≥3 risk factors	144 (113–184)	117 (102–133)	118	124 (112–138)	124 (110–138)	145 (126–167)	188 (154–230)	99 (91–108)
2 risk factors	99 (83–118)	88 (83–92)	87	82 (76–88)	90 (82–98)	101 (90–112)	114 (73–178)	69 (61–77)
≤1 risk factor	88 (77–101)	71 (62–80)	62	65 (61–69)	70 (64–77)	69 (62–77)	80 (67–93)	46 (44–49)
All-cause mortality in recent users								
≥3 risk factors	166 (121–230)	165 (131–207)	142	145 (126–165)	161 (135–189)	168 (146–193)	185 (158–215)	183 (146–229)
2 risk factors	147 (127–170)	123 (112–134)	104	104 (97–111)	108 (100–118)	104 (94–116)	130 (117–146)	131 (124–139)
≤1 risk factor	134 (113–157)	101 (93–109)	88	84 (78–89)	86 (75–99)	89 (81–98)	108 (96–121)	98 (85–111)

[a] In the primary cohort, among 101,649 prevalent users who initiated antihypertensive use before 2006, 17,979 (17.7%), 40,293 (39.6%), and 43,377 (42.7%) had ≥3, 2, and ≤1 risk factors, respectively. In the secondary cohort, among 220,105 prevalent users who initiated antihypertensive use before 2010, 48,785 (22.2%), 90,183 (40.9%), and 81,137 (36.9%) had ≥3, 2, and ≤1 risk factors, respectively.
[b] The summary effects and 95% CIs of the primary and secondary cohorts were calculated by using the DerSimonian-Laird random-effects model. The 1-year rates were estimated by multiplying the hazard ratios by the mean of the age specific rates in the reference group. All analyses were adjusted for age, sex, family history of cardiovascular disease, income level, smoking, alcohol consumption, exercise frequency, body mass index, diabetes, hyperlipidemia, and proteinuria.
[c] The time averaged systolic BP of 120–129 mm Hg served as the reference.
[d] The critical cardiorenal event was a composite of admission to critical care unit with cardiovascular or chronic kidney disease, revascularization for myocardial infarction or stroke, and new onset end-stage kidney disease.
BP, blood pressure.

© 2019 Jung HH. *JAMA Network Open.*

eTable 11. Yearly Event Rates After Further Adjustment for Compliance[a]

Outcome	Yearly Events (95% Confidence Interval) per 10000 Persons, in the Primary Cohort							
	Treated systolic BP, mm Hg							
	<110	110–119	120–129[b]	130–139	140–149	150–159	≥160	Missing case
Critical cardiorenal event[c]								
≥3 risk factors	195 (149–254)	139 (117–164)	140	159 (143–177)	165 (146–185)	183 (159–211)	229 (196–269)	147 (128–170)
2 risk factors	108 (87–135)	104 (93–118)	109	106 (98–115)	115 (105–125)	128 (115–143)	129 (114–147)	108 (98–120)
≤1 risk factor	106 (88–127)	92 (83–103)	81	84 (78–91)	95 (87–103)	94 (84–104)	102 (90–116)	74 (68–82)
All-cause mortality								
≥3 risk factors	263 (205–337)	247 (214–285)	192	207 (186–229)	226 (202–253)	235 (205–268)	289 (249–334)	325 (289–366)
2 risk factors	183 (154–218)	169 (152–187)	149	150 (139–161)	162 (149–176)	162 (146–179)	207 (186–231)	243 (224–263)
≤1 risk factor	191 (167–217)	141 (129–154)	123	122 (114–131)	131 (121–141)	137 (124–151)	160 (144–179)	196 (183–211)
**Outcome**	**Yearly Events (95% Confidence Interval) per 10000 Persons, in the Secondary Cohort**							
	**Treated systolic BP, mm Hg**							
	<110	110–119	120–129[b]	130–139	140–149	150–159	≥160	Missing case
Critical cardiorenal event[c]								
≥3 risk factors	198 (151–258)	158 (138–181)	161	170 (157–185)	174 (158–190)	200 (178–224)	236 (207–269)	158 (143–174)
2 risk factors	102 (83–126)	102 (92–113)	104	104 (97–112)	113 (103–123)	116 (102–132)	162 (139–188)	85 (78–92)
≤1 risk factor	91 (80–104)	63 (58–69)	65	68 (64–72)	73 (68–79)	80 (71–90)	107 (91–125)	43 (40–46)
All-cause mortality								
≥3 risk factors	270 (227–321)	257 (236–280)	232	229 (217–243)	221 (207–236)	230 (210–251)	282 (255–313)	251 (235–267)
2 risk factors	154 (134–178)	143 (132–156)	114	106 (99–113)	113 (104–123)	125 (111–142)	129 (109–153)	123 (114–131)
≤1 risk factor	83 (71–97)	60 (55–67)	53	56 (52–61)	60 (54–66)	66 (56–77)	88 (71–108)	59 (55–64)

[a] The 1-year rates were estimated by multiplying the hazard ratios by the mean of the age specific rates in the reference group. All analyses were adjusted for age, sex, family history of cardiovascular disease, income level, smoking, alcohol consumption, exercise frequency, body mass index, diabetes, hyperlipidemia, and proteinuria, and further adjusted for antihypertensive compliance.

[b] The time averaged systolic BP of 120–129 mm Hg served as the reference.

[c] The critical cardiorenal event was a composite of admission to critical care unit with cardiovascular or chronic kidney disease, revascularization for myocardial infarction or stroke, and new onset end-stage kidney disease.

BP, blood pressure.

© 2019 Jung HH. *JAMA Network Open.*

eTable 12. Yearly Event Rates in Risk Categories Grouped by Risk Factors After Exclusion of Proteinuria[a]

Outcome	Yearly Events (95% Confidence Interval) per 10000 Persons, in the Primary Cohort							
	Treated systolic BP, mm Hg							
	<110	110–119	120–129[b]	130–139	140–149	150–159	≥160	Missing case
Critical cardiorenal event[c]								
≥3 risk factors	204 (154–269)	140 (118–167)	137	153 (136–172)	153 (134–174)	175 (150–204)	227 (192–269)	114 (101–128)
2 risk factors	108 (86–135)	103 (91–116)	110	109 (101–118)	119 (109–129)	129 (116–143)	125 (110–142)	82 (76–88)
≤1 risk factor	108 (90–128)	95 (85–105)	83	84 (78–91)	94 (86–102)	94 (84–104)	103 (91–116)	58 (55–63)
All-cause mortality								
≥3 risk factors	258 (197–338)	253 (216–294)	185	205 (184–230)	223 (197–252)	239 (208–276)	298 (255–349)	266 (239–296)
2 risk factors	200 (169–237)	174 (157–193)	150	150 (140–162)	163 (151–177)	160 (145–176)	203 (182–225)	194 (181–207)
≤1 risk factor	203 (178–231)	145 (133–159)	125	122 (114–131)	130 (120–140)	137 (125–150)	161 (145–179)	148 (139–157)
**Outcome**	**Yearly Events (95% Confidence Interval) per 10000 Persons, in the Secondary Cohort**							
	**Treated systolic BP, mm Hg**							
	<110	110–119	120–129[b]	130–139	140–149	150–159	≥160	Missing case
Critical cardiorenal event[c]								
≥3 risk factors	116 (92–145)	99 (88–112)	105	109 (100–118)	127 (115–140)	139 (121–160)	177 (150–209)	100 (90–111)
2 risk factors	80 (67–96)	72 (66–79)	75	80 (75–85)	81 (75–87)	94 (85–105)	123 (108–139)	64 (60–69)
≤1 risk factor	81 (69–94)	57 (52–62)	57	59 (55–63)	64 (59–69)	66 (58–74)	84 (73–97)	41 (38–44)
All-cause mortality								
≥3 risk factors	119 (96–147)	131 (118–146)	111	115 (106–125)	124 (112–136)	136 (119–155)	159 (135–188)	132 (119–146)
2 risk factors	99 (85–116)	86 (80–94)	75	77 (73–82)	80 (74–86)	84 (75–93)	98 (87–111)	88 (83–94)
≤1 risk factor	89 (79–102)	70 (64–76)	63	60 (56–64)	60 (55–64)	64 (58–72)	78 (69–90)	65 (61–69)

[a] The risk categories were grouped by the number of risk factors present at baseline after exclusion of proteinuria: i.e., ≥3, 2, ≤1 of the four risk factors (hypertension, diabetes, hyperlipidemia, and smoking). Among a total of 487,412 primary cohort participants, 34,050 (7.0%), 110,023 (22.6%), and 343,339 (70.4%) had ≥3, 2, and ≤1 risk factors, respectively. Among a total of 915,563 secondary cohort participants, 65,631 (7.2%), 188,669 (20.6%), and 661,263 (72.2%) had ≥3, 2, and ≤1 risk factors, respectively. The 1-year rates were estimated by multiplying the hazard ratios by the mean of the age specific rates in the reference group. All analyses were adjusted for age, sex, family history of cardiovascular disease, income level, smoking, alcohol consumption, exercise frequency, body mass index, diabetes, hyperlipidemia, and proteinuria.

[b] The time averaged systolic BP of 120–129 mm Hg served as the reference.

[c] The critical cardiorenal event was a composite of admission to critical care unit with cardiovascular or chronic kidney disease, revascularization for myocardial infarction or stroke, and new onset end-stage kidney disease.

BP, blood pressure.

© 2019 Jung HH. *JAMA Network Open.*

eTable 13. Participation Rates in Health Screenings During The Study Periods

Year	No. of Survivors	Participants in Health Screening
	**Primary Cohort**	
2002–2005	487412	487412 (100.0%)
2006–2007	481733	345602 (71.7%)
2008–2009	475264	364332 (76.7%)
2010–2011	467908	366726 (78.4%)
2012–2013	459581	352550 (76.7%)
2014–2015	451039	352732 (78.2%)
	**Secondary Cohort**	
2006–2008	915563	627615 (68.5%)
2009–2010	915563	915563 (100.0%)
2011–2012	901111	737015 (81.8%)
2013–2014	891280	715404 (80.3%)
2015–2016	879436	704835 (80.1%)

© 2019 Jung HH. *JAMA Network Open.*

eReferences

1. Cheol Seong S, Kim Y-Y, Khang Y-H, et al. Data Resource Profile: The National Health Information Database of the National Health Insurance Service in South Korea. *Int J Epidemiol*. 2017;46(3):799-800.

2. Lee J, Lee JS, Park S-H, Shin SA, Kim K. Cohort Profile: The National Health Insurance Service-National Sample Cohort (NHIS-NSC), South Korea. *Int J Epidemiol*. 2017;46(2):e15.

3. Jung HH, Park JI, Jeong JS. Blood Pressure-Related Risk Among Users Versus Nonusers of Antihypertensives: A Population-Based Cohort in Korea. *Hypertension*. 2018;71(6):1047-1055.

4. Conroy RM, Pyörälä K, Fitzgerald AP, et al. Estimation of ten-year risk of fatal cardiovascular disease in Europe: the SCORE project. *Eur Heart J*. 2003;24(11):987-1003.

5. Collins D, Lee J, Bobrovitz N, Koshiaris C, Ward A, Heneghan C. whoishRisk - an R package to calculate WHO/ISH cardiovascular risk scores for all epidemiological subregions of the world. *F1000Res*. 2016;5:2522.

6. Jung KJ, Jang Y, Oh DJ, et al. The ACC/AHA 2013 pooled cohort equations compared to a Korean Risk Prediction Model for atherosclerotic cardiovascular disease. *Atherosclerosis*. 2015;242(1):367-375.

7. D'Agostino RB Sr, Vasan RS, Pencina MJ, et al. General cardiovascular risk profile for use in primary care: the Framingham Heart Study. *Circulation*. 2008;117(6):743-753.

8. Kimm H, Yun JE, Lee S-H, Jang Y, Jee SH. Validity of the diagnosis of acute myocardial infarction in korean national medical health insurance claims data: the korean heart study (1). *Korean Circ J*. 2012;42(1):10-15.

© 2019 Jung HH. *JAMA Network Open.*
